# Supplementary material for: TGF‐β1 secreted by Tregs in lymph nodes promotes breast cancer malignancy via up‐regulation of IL‐17RB
Source: EMBO Mol Med. 2017 Oct 9;9(12):1660–80. doi: 10.15252/emmm.201606914 (PMC5709760; doi:10.15252/emmm.201606914)
Supplement: Supplementary file 1 — Appendix [file EMMM-9-1660-s001.pdf]

Appendix

Table of content

Appendix Figure S1

Appendix Figure S2

Appendix Figure S3

Appendix Figure S4

Appendix Figure S5

Appendix Figure S6

Appendix Figure S7

Appendix Table S1

Appendix Table S2

Appendix Table S3

Appendix Figure S1

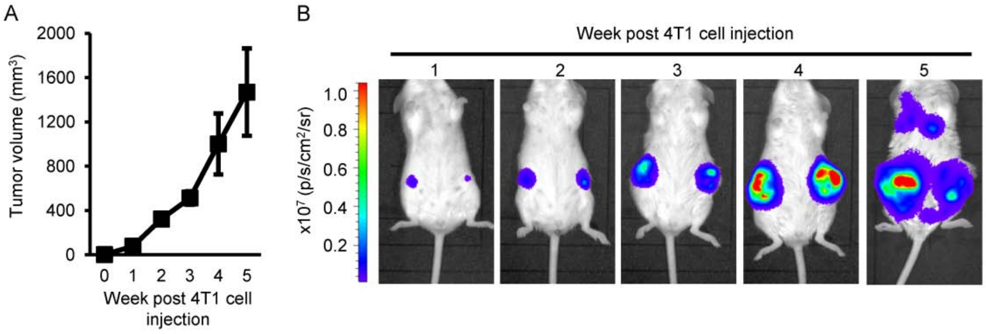

Appendix Figure S1. Kinetics of distant organ metastasis in 4T1 tumor-bearing mice.

(A) Tumor growth cruve in BALB/c mice injected with 4T1 cells. $1x10^6$ 4T1 cells were injected into the fourth mammary fat pad on the both side and the tumor volumes were evaluated weekly.

(B) Representative IVIS images of the 4T1 tumor-bearing mice. The bioluminescent signal (pseudocolor) was recorded as photons per second per centimeter squared per steradian ($p/s/cm^2/sr$). The luminescent image was overlaid on the photographic image.

Data information: In (A), data are presented as mean ± SD (n = 3 mice per time point).

Appendix Figure S2

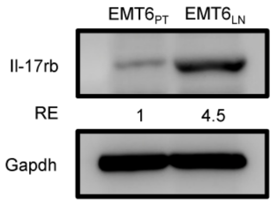

Appendix Figure S2. Il-17rb expression in $EMT6_{PT}$ and $EMT6_{LN}$ cells.

Il-17rb expression in $EMT6_{PT}$ and $EMT6_{LN}$ cells were examined by Western blotting analysis. The intensity of each band was quantified using the Image J software, and Gapdh was used as a loading control. Relative expression (RE) of Il-17rb levels in $EMT6_{LN}$ cells to $EMT6_{PT}$ cells is indicated.

Appendix Figure S3

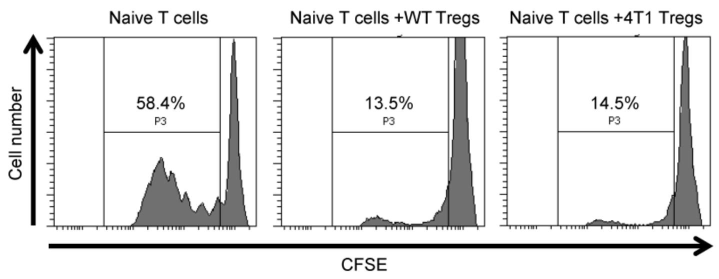

Appendix Figure S3. Tregs isolated from inguinal LNs in 4T1 tumor-bearing mice exhibit similar T cell suppressive activity to Tregs isolated from WT mice. Tregs ($CD4^+CD25^+GITR^+$cells) were isolated from the inguinal LNs of WT mice (WT Tregs) and from the 4T1 tumor-bearing mice at the third week after initial injection (4T1 Tregs) by FACS sorting. $5x10^4$ Tregs were then co-cultured with $1x10^5$ CFSE-labeled $CD3^+$ T cells (naive T cells) from WT mice in the 96-well U bottom plate pre-coated with anti-mouse CD3 Abs and anti-mouse CD28 Abs. Five days later, the percentage of proliferating CFSE-labeled $CD3^+$ T cells were analyzed by FACS.

Appendix Figure S4

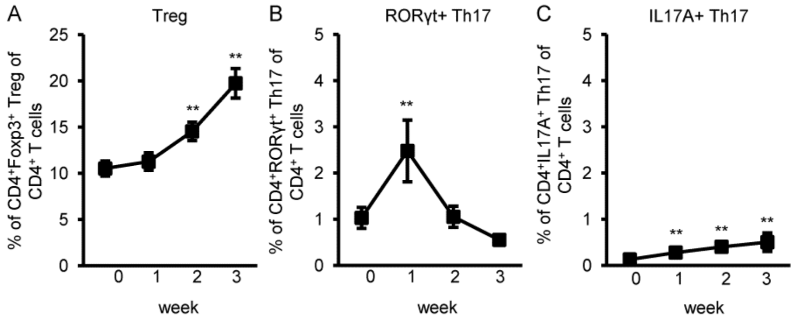

Appendix Figure S4. Percentage of Tregs and Th17 cells in inguinal LN of 4T1 tumor-bearing mice.

Percentage of $CD4^+Foxp3^+$ Tregs (A), $CD4^+ROR\gamma t^+$ Th17 cells (B), or $CD4^+IL\text{-}17A^+$ Th17 cells (C) of total $CD4^+$ cells in inguinal LN of BALB/c mice injected with 4T1 cells was analyzed by FACS.

Data information: All values are presented as mean ± SD (n = 4 mice per group). In (A), **P = 0.0017 and **P = 0.0001 for the percentage of $CD4^+Foxp3^+$ Tregs at week 2 and week 3, respectively. In (B), **P = 0.0012 for the percentage of $CD4^+ROR\gamma t^+$ Th17 cells at week 1. In (C), **P = 0.0054, **P = 0.0012, and **P = 0.0109 for the percentage of $CD4^+IL\text{-}17A^+$ Th17 cells at week 1, 2, and 3, respectively. Level of significance was determined using two-tailed unpaired t-test.

Appendix Figure S5

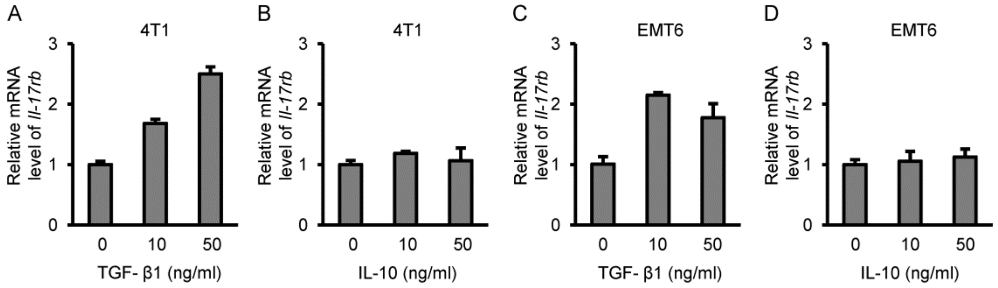

Appendix Figure S5. TGF-β1, but not IL-10, induces Il-17rb expression in 4T1 and EMT6 breast cancer cells.

RT-qPCR analysis of *Il-17rb* in 4T1 cells (A and B) or EMT6 cells (C and D) treated with recombinant TGF-β1 (A and C) or IL-10 (B and D) for 4 hours. Gapdh was used as internal control.

Data information: All experimental data were verified in at least two independent experiments. Data were presented as means ± SD (triplicate measurement).

Appendix Figure S6

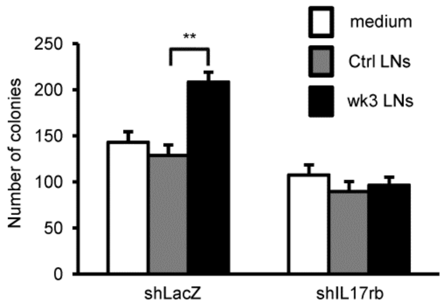

Appendix Figure S6. Depletion of Il-17rb abolishes the colony forming ability induced by the tumor-draining LNs.

4T1-injected BALB/c mice were sacrificed at the third week after initial injection. Total cells isolated from inguinal lymph node tissues were co-cultured with shLacZ or shIl-17rb 4T1 cells in a transwell plate ($5x10^2$ cells/well, *n* = 6 wells per group). Inguinal lymph node tissues from un-injected BALB/c mice were used as a control. After 5-day co-culture, the colony forming ability of 4T1 cells at lower wells was examined in soft agar.

Data information: Data were presented as means ± SD. (*n* = 6 wells per group, **P = 0.0000012). Level of significance was determined using two-tailed unpaired t-test.

Appendix Figure S7

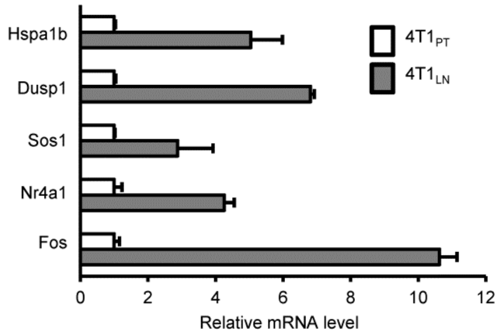

Appendix Figure S7. Gene expression of *Fos, Nr4a1, Sos1, Dusp1,* and *Hspa1b* involved in MAPK signaling pathway is increased in $4T1_{LN}$ cells.

mRNA expression of each candidate gene in $4T1_{PT}$ and $4T1_{LN}$ cells was determined by RT-qPCR. Gapdh was used as an internal control.

Data information: Data were presented as means ± SD (triplicate measurement).

Appendix Table S1. KEGG pathway enrichment analysis of the up-regulated genes in $4T1_{LN}$ cells.

Term	*P* value	Genes
MAPK signaling pathway	0.033	Fgf16,Fos,Nr4a1,Sos1,Dusp1, Hspa1b
PI3K-Akt signaling pathway	0.038	Fgf16,Nr4a1,Tnxb,Sos1,Cdc37, Itga10,Thbs2
Prolactin signaling pathway	0.095	Fos, Sos1, Socs3

Appendix Table S2. KEGG pathway enrichment analysis of the down-regulated genes in shIL-17RB MDA-MB-361 cells.

Term	*P* value	Genes
Transcriptional misregulation in cancer	0.073	DDIT3,ETV4,SUPT3H,ARNT2, MMP9
Pathways in cancer	0.075	MECOM,WNT3,ARNT2,FGF1, LAMA2,MMP1, MMP9,PDGFRB
MAPK signaling pathway	0.091	DDOT3,MECOM,DUSP4,FGF1, PDGFRB,PTPRR
Staphylococcus aureus infection	0.091	C1R,C5,HLA-DRA
Legionellosis	0.091	CXCL2,CXCL3,EEF1A2

Appendix Table S3. Primers used in this study.

a. Primer sequence for real-time PCR.

Gene	Forward	Reverse
Il-17rb	5'-tggtctatcttgggggagca-3'	5'-aaagctgtggcgtccttcat-3'
Cd300Ig	5'-gaccgtgatcatgagggacc-3'	5'-accaggagaagctggaaagac-3'
Gpr56	5'-ctgcctgggctctatctact-3'	5'-ctgggtctctgggtaagagtg-3'
Scara5	5'-gttccatgatcgtcgttggg-3'	5'-ccaaatcctccctgtgcctt-3'
Oprk1	5'-caagtgccaccttctcgctt-3'	5'-ctgtcggattctgcccagtt-3'
GAPDH	5'-ctttggcattgtggaagggc-3'	5'-cagggatgatgttctgggca-3'
Hspa1b	5'-caccatcgaggaggtggattag-3'	5'-ttgacagtaatcggtgcccaa-3'
Dusp1	5'-gcttgacacacccaccagta-3'	5'-cagaccaccgacctacacaa-3'
Sos1	5'-agccagtgcagcaaaacttg-3'	5'-ggaactccctttgtgagcca-3'
Nr4a1	5'-gagttcggcaagcctaccat-3'	5'-ggtgtcaaactctccggtgt-3'
Fos	5'-tactaccattccccagccga-3'	5'-gctgtcaccgtggggataaa-3'

b. Primers used for the cloning of Il-17rb into pLAS5w.Pbsd-L-tRFP-C vector.

Gene	Forward	Reverse
Il-17rb	5'-gatcttcgaaatgttgctagtgttgctgat-3'	5'-aagcgctagcctacaagggtgaacagctat-3'

c. Primers used for the detection of chromosomal deletions of Il-17rb.

	Forward	Reverse
Primer	5'-actctgactgcttgtgtttgt-3'	5'-tgggccggaaacaggagtat-3'

d. Primer used for the sequencing analysis of chromosomal deletions of Il-17rb.

	Forward	Reverse
Primer	5'-gattccttttgctcttgggc-3'	5'-tgaacaggtgatggaacaggag-3'
