# Supplementary material for: TGF‐β1 secreted by Tregs in lymph nodes promotes breast cancer malignancy via up‐regulation of IL‐17RB
Source: EMBO Mol Med. 2017 Oct 9;9(12):1660–80. doi: 10.15252/emmm.201606914 (PMC5709760; doi:10.15252/emmm.201606914)
Supplement: Supplementary file 2 — Expanded View Figures PDF [file EMMM-9-1660-s002.pdf]

# Expanded View Figures

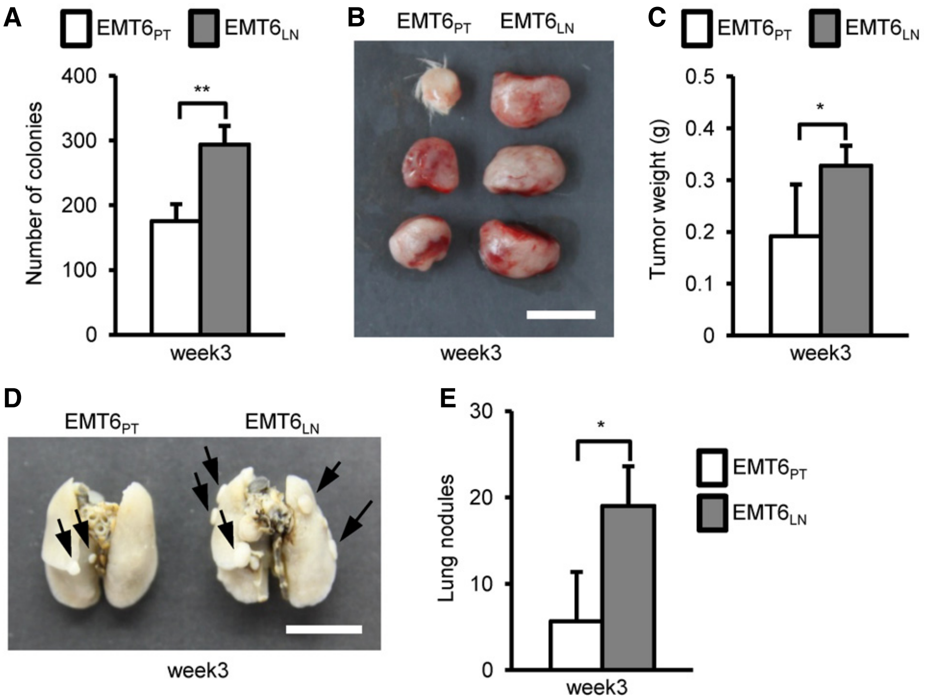


**Figure EV1. EMT6 breast cancer cells derived from tumor-draining lymph nodes gain aggressive malignancy.**

EMT6-injected mice were sacrificed at the third week after initial injection. Collagenase-digested specimens from the primary tumors or inguinal LN tissues were cultured in culture dishes. The detailed process is described in the methods section. After 3–5 days culture, viable $EMT6_{PT}$ and $EMT6_{LN}$ cells were used for the following assays.

A Soft-agar colony-forming activity was examined in $EMT6_{PT}$ and $EMT6_{LN}$ cells ($4 \times 10^2$ cells/well, $n$ = 4 wells per group).

B, C Tumorigenesis assays were determined in BALB/c mice ($n$ = 3 mice per group) orthotopically injected with $1 \times 10^2$ $EMT6_{PT}$ or $EMT6_{LN}$ cells. Tumor mass (B) and tumor weight (C) were measured on day 28. Scale bar: 1 cm.

D, E BALB/c mice ($n$ = 3 mice per group) were injected with $1 \times 10^5$ $EMT6_{PT}$ or $EMT6_{LN}$ cells via tail vein. Lung colonization was examined by lung morphology (D) and the numbers of tumor nodule (E) on day 21. Scale bar: 1 cm. Black arrow: Lung nodule.

Data information: All experimental data were verified in at least two independent experiments. All values are presented as mean ± SD. In (A), $**P = 0.00002$. In (C), $*P = 0.045788$. In (E), $*P = 0.034109$. Level of significance was determined using one-tailed (C) or two-tailed (A, E) unpaired $t$-test.

 © 2017 The Authors

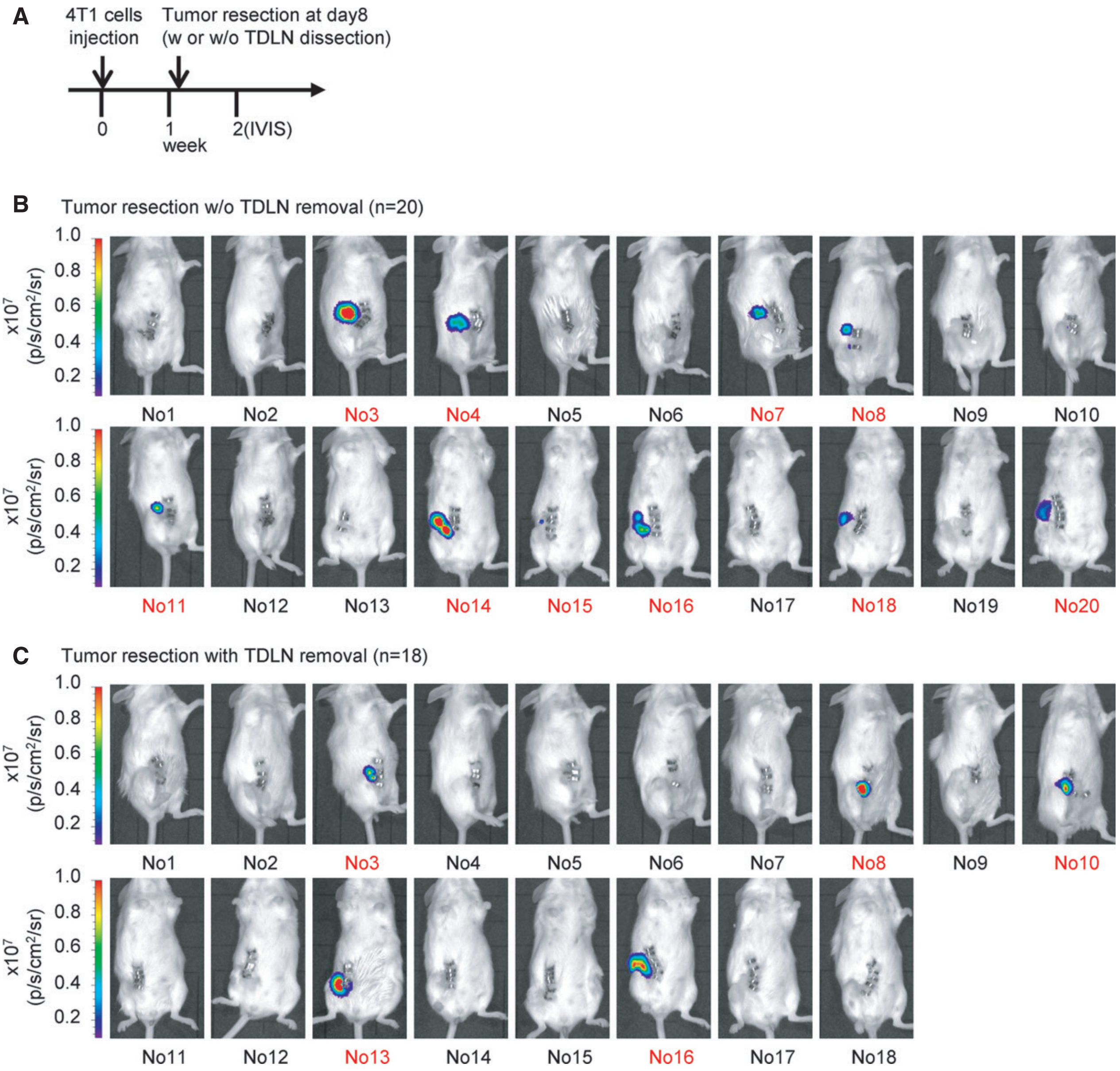


**Figure EV2. Bioluminescent images of tumor-bearing mice after tumor resection with or without tumor-draining lymph node dissection are taken at week 2.**

A Schematic diagram of tumor resection with or without tumor-draining lymph node removal in 4T1 tumor-bearing mice.

B, C The recurrence of primary tumor was examined by bioluminescent images of tumor-bearing mice after tumor resection on week 2. Ten control mice (surgical removal of a primary tumor) and five experimental mice (surgical removal of both primary tumor and inguinal LN) were excluded due to primary tumor recurrence at week 2. The excluded mice are labeled in red.

© 2017 The Authors

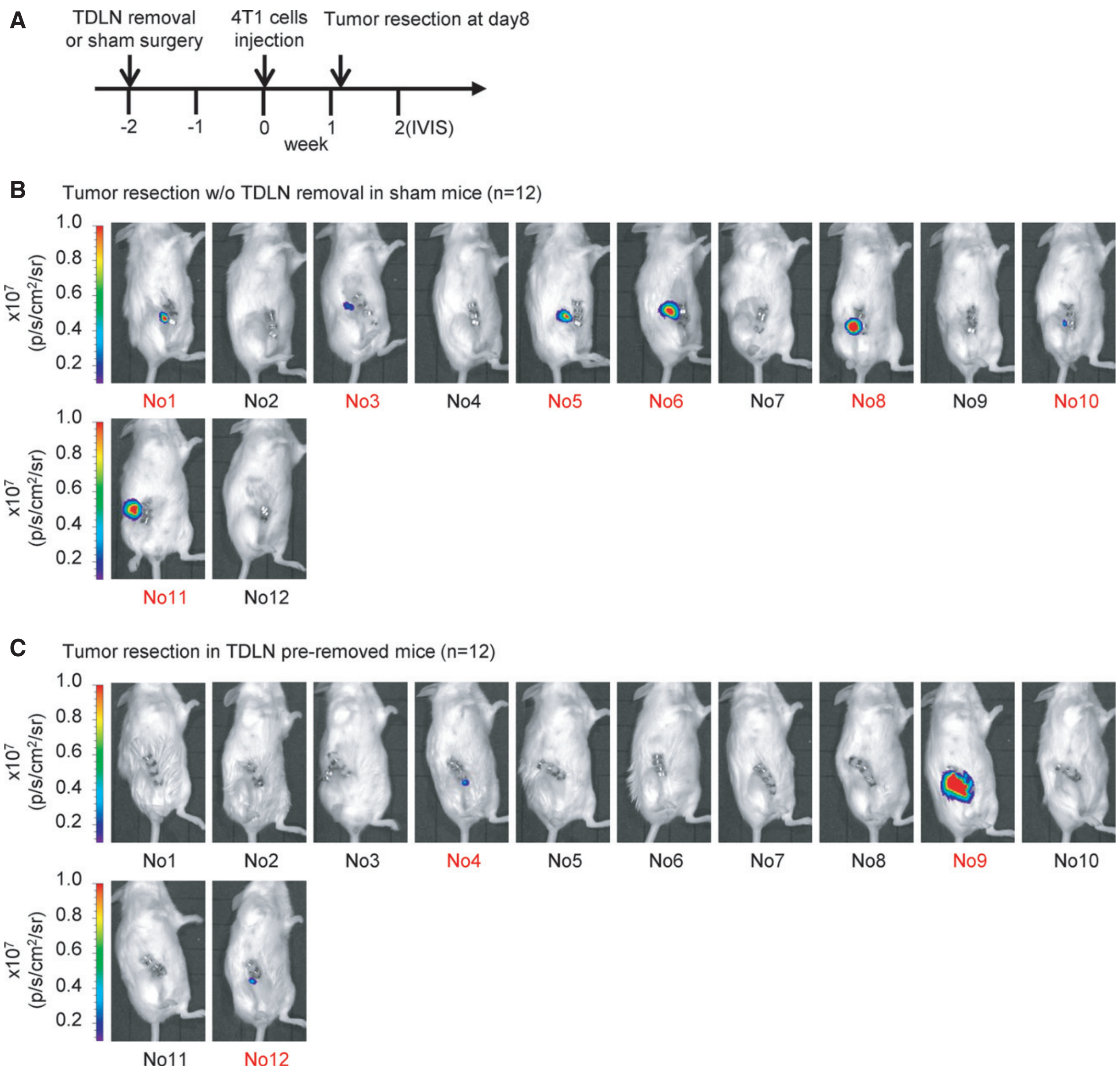


**Figure EV3. Bioluminescent images of inguinal lymph node pre-removed tumor-bearing mice or sham surgery tumor-bearing mice after tumor resection are taken at week 2.**

A Schematic diagram of tumor resection in inguinal lymph node pre-removed 4T1 tumor-bearing mice or sham surgery tumor-bearing mice.

B, C The recurrence of primary tumor was examined by bioluminescent images of tumor-bearing mice after tumor resection on week 2. Seven control mice (surgical removal of a primary tumor in sham control surgery mice) and three experimental mice (surgical removal of a primary tumor in lymph node-removed mice) are excluded due to the primary tumor recurrence at week 2 and labeled in red.

 © 2017 The Authors

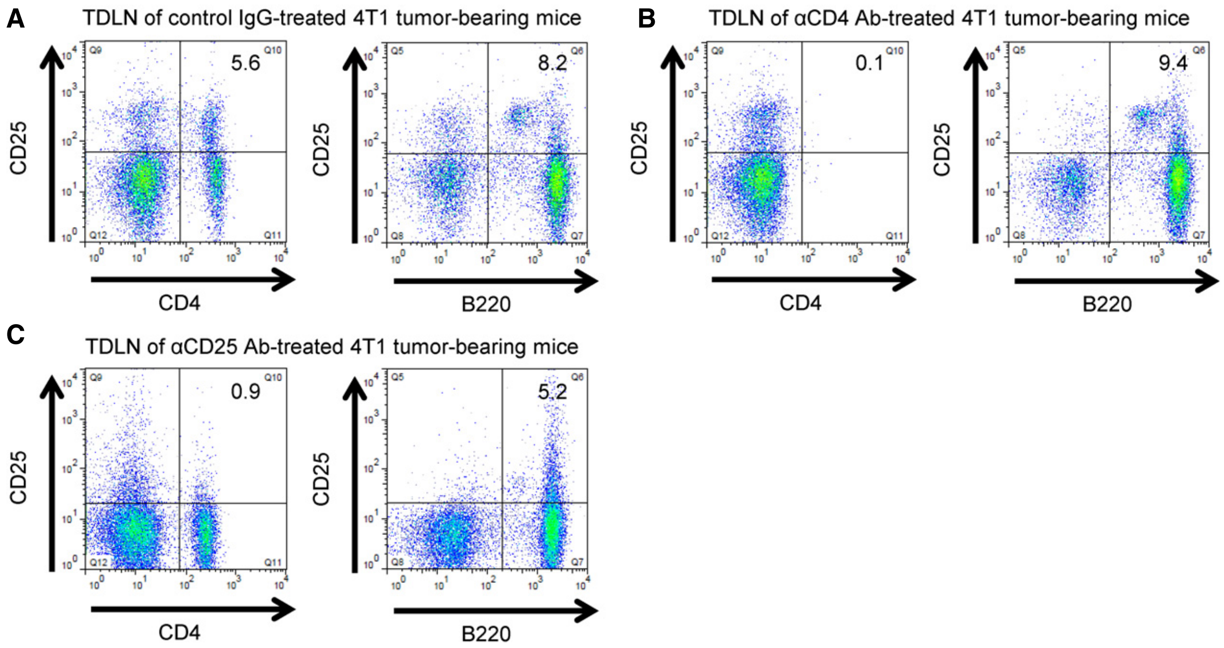


**Figure EV4. Injection of anti-CD4 or anti-CD25 neutralizing antibody into 4T1 tumor-bearing mice induces the depletion of Tregs, but not Bregs.**

A–C 4T1-injected BALB/c mice were treated with control IgG (A), anti-CD4 (B), or anti-CD25 (C) neutralizing antibody at the day 15 and day 18 after initial 4T1 cells injection. Mice were sacrificed at 3 weeks after initial injection. The $CD4^{+}CD25^{+}$ Treg population (left panel), and $B220^{+}CD25^{+}$ Breg (right panel) population in inguinal LNs were confirmed by FACS analysis.

© 2017 The Authors 

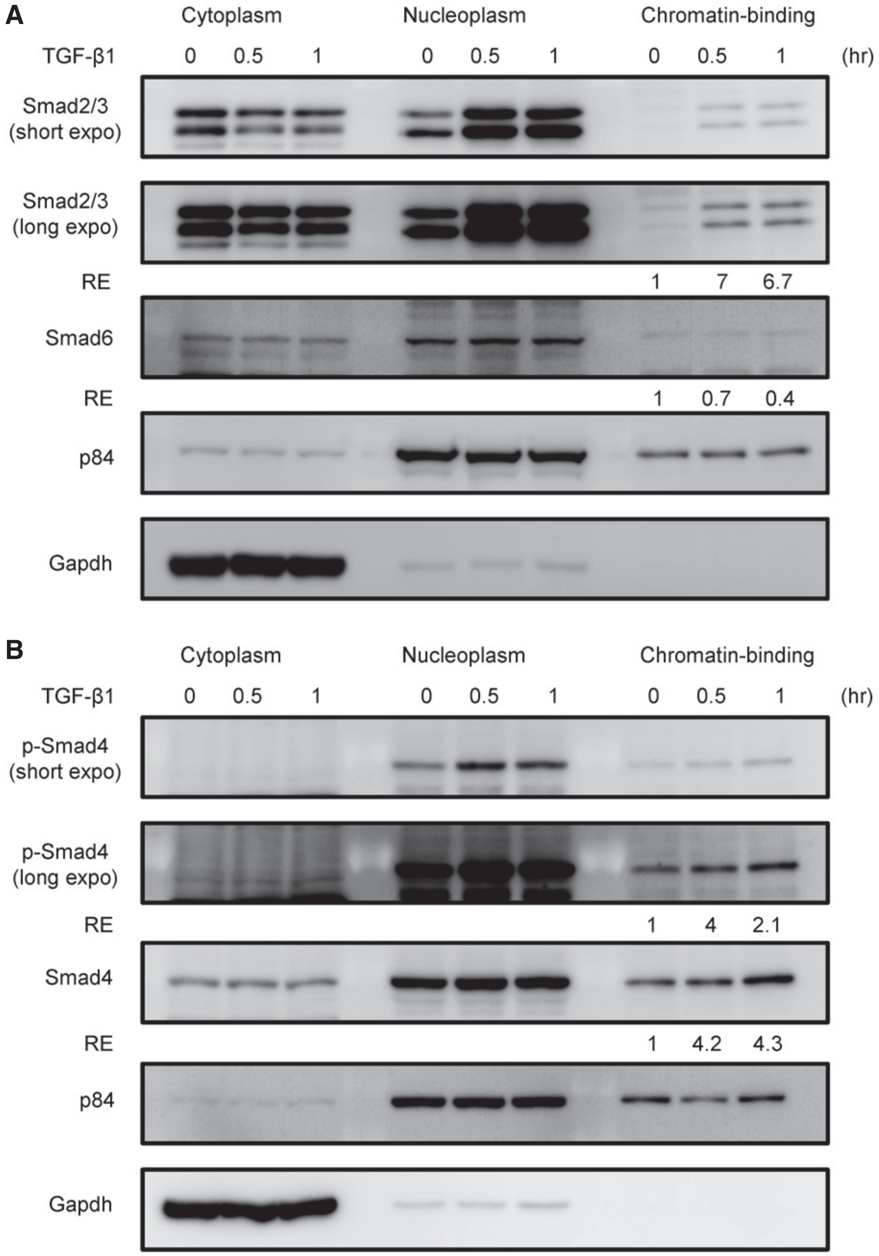


**Figure EV5. TGF-β1 treatment induces the chromatin binding of Smad2/3/4 in 4T1 cells.**

A, B Western blotting analyzed Smads in 4T1 cells treated with recombinant TGF-β1 proteins. After treatment, the cytoplasm-, nucleoplasm-, and chromatin-binding fractions of TGFβ-treated 4T1 cells were isolated, respectively, by using commercial sub-cellular protein fractionation kit for cultured cells (Thermo Scientific #78840). All experimental data were verified in at least two independent experiments. The intensity of each band was quantified using the ImageJ software. Gapdh or p84 was used as a loading control. Relative expression (RE) of protein levels in each sample to control untreated 4T1 cells is indicated.

Source data are available online for this figure.


© 2017 The Authors
