# Supplementary material for: TGF‐β1 secreted by Tregs in lymph nodes promotes breast cancer malignancy via up‐regulation of IL‐17RB
Source: EMBO Mol Med. 2017 Oct 9;9(12):1660–80. doi: 10.15252/emmm.201606914 (PMC5709760; doi:10.15252/emmm.201606914)
Supplement: Supplementary file 3 — Table EV1 [file EMMM-9-1660-s003.docx]

**Table EV1. The up-regulated genes in 4T1_LN_ cells, compared to 4T1_PT_ cells, as determined by microarray analysis.**

| Gene | Description | 4T1_LN_/  4T1_PT_ (log_2_) |
| --- | --- | --- |
| Hcn1 | hyperpolarization-activated, cyclic nucleotide-gated K+ 1 | 9.98 |
| Ccdc58 | coiled-coil domain containing 58 | 8.26 |
| Zfp346 | zinc finger protein 346 | 6.78 |
| Trpc3 | transient receptor potential cation channel, subfamily C, member 3 | 6.66 |
| Mobp | myelin-associated oligodendrocytic basic protein | 6.66 |
| Camk4 | calcium/calmodulin-dependent protein kinase IV | 4.97 |
| Fam101a | family with sequence similarity 101, member A | 4.81 |
| Cd109 | CD109 antigen | 4.74 |
| Zscan29 | zinc finger SCAN domains 29 | 4.72 |
| Slco4a1 | solute carrier organic anion transporter family, member 4a1 | 4.69 |
| Ccdc74a | coiled-coil domain containing 74A | 4.62 |
| Bhmt | betaine-homocysteine methyltransferase | 4.60 |
| Chdh | choline dehydrogenase | 4.48 |
| Spint2 | serine protease inhibitor, Kunitz type 2 | 4.44 |
| Ankrd61 | ankyrin repeat domain 61 | 4.31 |
| Smarcd1 | SWI/SNF related, matrix associated, actin dependent regulator of chromatin, subfamily d, member 1 | 4.05 |
| Mest | mesoderm specific transcript | 3.96 |
| Hey1 | hairy/enhancer-of-split related with YRPW motif 1 | 3.76 |
| Slc26a6 | solute carrier family 26, member 6 | 3.71 |
| Id4 | inhibitor of DNA binding 4 | 3.70 |
| Gata2 | GATA binding protein 2 | 3.50 |
| Sos1 | son of sevenless homolog 1 (Drosophila) | 3.38 |
| Dclk1 | doublecortin-like kinase 1 | 3.13 |
| Serinc5 | serine incorporator 5 | 3.01 |
| Ucma | upper zone of growth plate and cartilage matrix associated | 2.92 |
| Magea1  \|Magea6 | melanoma antigen, family A, 1\|melanoma antigen, family A, 6 | 2.84 |
| Gem | GTP binding protein (gene overexpressed in skeletal muscle) | 2.83 |
| Dclre1c | DNA cross-link repair 1C, PSO2 homolog (S. cerevisiae) | 2.80 |
| Olfr1510 | olfactory receptor 1510 | 2.78 |
| Tnxb | tenascin XB | 2.72 |
| Nr4a1 | nuclear receptor subfamily 4, group A, member 1 | 2.72 |
| Gtf2a2 | general transcription factor II A, 2 | 2.70 |
| Cdh20 | cadherin 20 | 2.66 |
| Il-17rb | interleukin 17 receptor B | 2.63 |
| Prom2 | prominin 2 | 2.61 |
| Cd300lg | CD300 antigen like family member G | 2.56 |
| Fxyd3 | FXYD domain-containing ion transport regulator 3 | 2.50 |
| Plp1 | proteolipid protein (myelin) 1 | 2.49 |
| Slitrk4 | SLIT and NTRK-like family, member 4 | 2.49 |
| Gpr56 | G protein-coupled receptor 56 | 2.48 |
| Hs3st1 | heparan sulfate (glucosamine) 3-O-sulfotransferase 1 | 2.44 |
| Cd59a | CD59a antigen | 2.44 |
| Slc14a1 | solute carrier family 14 (urea transporter), member 1 | 2.41 |
| Scara5 | scavenger receptor class A, member 5 (putative) | 2.40 |
| AI646023 | expressed sequence AI646023 | 2.39 |
| Olfm1 | olfactomedin 1 | 2.36 |
| Zfp36 | zinc finger protein 36 | 2.29 |
| Olfr1443 | olfactory receptor 1443 | 2.28 |
| Oprk1 | opioid receptor, kappa 1 | 2.27 |
| Dyrk1b | dual-specificity tyrosine-(Y)-phosphorylation regulated kinase 1b | 2.26 |
| Glb1l | galactosidase, beta 1-like | 2.21 |
| Pgap1 | post-GPI attachment to proteins 1 | 2.21 |
| Rnf128 | ring finger protein 128 | 2.18 |
| Mon1a | MON1 homolog A (yeast) | 2.13 |
| Rasgef1b | RasGEF domain family, member 1B | 2.12 |
| Cyp24a1 | cytochrome P450, family 24, subfamily a, polypeptide 1 | 2.12 |
| Trappc9 | trafficking protein particle complex 9 | 2.12 |
| Als2 | amyotrophic lateral sclerosis 2 (juvenile) homolog (human) | 2.10 |
| Sybu | syntabulin (syntaxin-interacting) | 2.10 |
| Esyt3 | extended synaptotagmin-like protein 3 | 2.06 |
| Chpt1 | choline phosphotransferase 1 | 2.05 |
| Otop1 | otopetrin 1 | 2.05 |
| Hspa1b | heat shock protein 1B | 2.01 |
| Aqp5 | aquaporin 5 | 2.01 |
| Ptgs1 | prostaglandin-endoperoxide synthase 1 | 2.00 |
| Thbs2 | thrombospondin 2 | 1.98 |
| Mafb | v-maf musculoaponeurotic fibrosarcoma oncogene family, protein B (avian) | 1.97 |
| Defb37 | defensin beta 37 | 1.97 |
| Hmgcr | 3-hydroxy-3-methylglutaryl-Coenzyme A reductase | 1.96 |
| Notch1 | Notch gene homolog 1 (Drosophila) | 1.93 |
| Tmem138 | transmembrane protein 138 | 1.90 |
| Fos | FBJ osteosarcoma oncogene | 1.90 |
| Cdadc1 | cytidine and dCMP deaminase domain containing 1 | 1.87 |
| Snord118 | small nucleolar RNA, C/D box 118 | 1.86 |
| Dhx8 | DEAH (Asp-Glu-Ala-His) box polypeptide 8 | 1.85 |
| Klf2 | Kruppel-like factor 2 (lung) | 1.85 |
| Zfp518a | zinc finger protein 518A | 1.84 |
| D0H4S114 | DNA segment, human D4S114 | 1.84 |
| Zmym3 | zinc finger, MYM-type 3 | 1.84 |
| Nr4a2 | nuclear receptor subfamily 4, group A, member 2 | 1.82 |
| Stxbp6 | syntaxin binding protein 6 (amisyn) | 1.82 |
| Zfml | zinc finger, matrin-like | 1.81 |
| Pvrl3 | poliovirus receptor-related 3 | 1.81 |
| Olfr561 | olfactory receptor 561 | 1.80 |
| Lgals2 | lectin, galactose-binding, soluble 2 | 1.79 |
| Hist1h4i | histone cluster 1, H4i | 1.79 |
| Dusp1 | dual specificity phosphatase 1 | 1.79 |
| Cyp26b1 | cytochrome P450, family 26, subfamily b, polypeptide 1 | 1.78 |
| Aplnr | apelin receptor | 1.78 |
| Aqp1 | aquaporin 1 | 1.76 |
| Crlf1 | cytokine receptor-like factor 1 | 1.75 |
| Klk10 | kallikrein related-peptidase 10 | 1.74 |
| Baz2b | bromodomain adjacent to zinc finger domain, 2B | 1.74 |
| Ndrg2 | N-myc downstream regulated gene 2 | 1.73 |
| Ano1 | anoctamin 1, calcium activated chloride channel | 1.72 |
| Arrdc3 | arrestin domain containing 3 | 1.72 |
| Orai3 | ORAI calcium release-activated calcium modulator 3 | 1.72 |
| Lactb | lactamase, beta | 1.71 |
| Pcolce2 | procollagen C-endopeptidase enhancer 2 | 1.71 |
| Ankrd55 | ankyrin repeat domain 55 | 1.71 |
| Zfp760 | zinc finger protein 760 | 1.70 |
| Itga10 | integrin, alpha 10 | 1.70 |
| Aldh3a1 | aldehyde dehydrogenase family 3, subfamily A1 | 1.70 |
| Selp | selectin, platelet | 1.69 |
| Rhoj | ras homolog gene family, member J | 1.68 |
| Bpifb1 | BPI fold containing family B, member 1 | 1.68 |
| Id2 | inhibitor of DNA binding 2 | 1.68 |
| Rbpms | RNA binding protein gene with multiple splicing | 1.67 |
| Id1 | inhibitor of DNA binding 1 | 1.67 |
| Grk4 | G protein-coupled receptor kinase 4 | 1.67 |
| Cenpt | centromere protein T | 1.67 |
| Fam131a | family with sequence similarity 131, member A | 1.66 |
| Pcm1 | pericentriolar material 1 | 1.65 |
| Ndrg4 | N-myc downstream regulated gene 4 | 1.65 |
| Cpne2 | copine II | 1.65 |
| Gpr97 | G protein-coupled receptor 97 | 1.64 |
| H19 | H19 fetal liver mRNA | 1.64 |
| Wbp1 | WW domain binding protein 1 | 1.64 |
| Ace | angiotensin I converting enzyme (peptidyl-dipeptidase A) 1 | 1.63 |
| Sema7a | sema domain, immunoglobulin domain (Ig), and GPI membrane anchor, (semaphorin) 7A | 1.63 |
| Cdc37 | cell division cycle 37 homolog (S. cerevisiae) | 1.63 |
| Nrp1 | neuropilin 1 | 1.62 |
| Mest | mesoderm specific transcript | 1.61 |
| Ddx1 | DEAD (Asp-Glu-Ala-Asp) box polypeptide 1 | 1.60 |
| Lin9 | lin-9 homolog (C. elegans) | 1.60 |
| Hist1h1c | histone cluster 1, H1c | 1.59 |
| Lig3 | ligase III, DNA, ATP-dependent | 1.59 |
| Nptx1 | neuronal pentraxin 1 | 1.59 |
| Fgf16 | fibroblast growth factor 16 | 1.57 |
| Socs3 | suppressor of cytokine signaling 3 | 1.56 |
| Accn2 | amiloride-sensitive cation channel 2, neuronal | 1.55 |
| Lgr4 | leucine-rich repeat-containing G protein-coupled receptor 4 | 1.55 |
| Irx2 | Iroquois related homeobox 2 (Drosophila) | 1.55 |
| Rtn2 | reticulon 2 (Z-band associated protein) | 1.55 |
| Mnat1 | menage a trois 1 | 1.54 |
| Fastkd2 | FAST kinase domains 2 | 1.54 |
| Vdr | vitamin D receptor | 1.53 |
| Adamts9 | a disintegrin-like and metallopeptidase (reprolysin type) with thrombospondin type 1 motif, 9 | 1.53 |
| Mllt3 | myeloid/lymphoid or mixed-lineage leukemia (trithorax homolog, Drosophila); translocated to, 3 | 1.52 |
| Ebpl | emopamil binding protein-like | 1.52 |
| Hist1h4m | histone cluster 1, H4m | 1.51 |
| Fbln2 | fibulin 2 | 1.51 |
| Adh7 | alcohol dehydrogenase 7 (class IV), mu or sigma polypeptide | 1.50 |
