# Supplementary material for: TGF‐β1 secreted by Tregs in lymph nodes promotes breast cancer malignancy via up‐regulation of IL‐17RB
Source: EMBO Mol Med. 2017 Oct 9;9(12):1660–80. doi: 10.15252/emmm.201606914 (PMC5709760; doi:10.15252/emmm.201606914)
Supplement: Supplementary file 4 — Table EV2 [file EMMM-9-1660-s004.docx]

**Table EV2. The down-regulated genes in 4T1_LN_ cells, compared to 4T1_PT_ cells, as determined by microarray analysis.**

| Gene | Description | 4T1_LN_/  4T1_PT_ (log_2_) |
| --- | --- | --- |
| Eid1 | EP300 interacting inhibitor of differentiation 1 | -11.47 |
| Rsad2 | radical S-adenosyl methionine domain containing 2 | -9.58 |
| Nomo1 | nodal modulator 1 | -8.92 |
| Ttc39a | tetratricopeptide repeat domain 39A | -7.47 |
| Rergl | RERG/RAS-like | -6.70 |
| Kcnj6 | potassium inwardly-rectifying channel, subfamily J, member 6 | -6.64 |
| Olfr663 | olfactory receptor 663 | -6.30 |
| Lamc2 | laminin, gamma 2 | -5.68 |
| Ccl2 | chemokine (C-C motif) ligand 2 | -5.52 |
| Rhox5 | reproductive homeobox 5 | -5.48 |
| Ppp1r16b | protein phosphatase 1, regulatory (inhibitor) subunit 16B | -4.65 |
| Vill | villin-like | -4.58 |
| Steap4 | STEAP family member 4 | -4.47 |
| Cfb | complement factor B | -4.30 |
| Ppp1r17 | protein phosphatase 1, regulatory subunit 17 | -4.09 |
| Cyp3a59  \|Cyp3a25 | cytochrome P450, subfamily 3A, polypeptide 59\|cytochrome P450, family 3, subfamily a, polypeptide 25 | -4.08 |
| Senp3 | SUMO/sentrin specific peptidase 3 | -4.08 |
| Smoc1 | SPARC related modular calcium binding 1 | -4.08 |
| Krt78 | keratin 78 | -4.07 |
| Irg1 | immunoresponsive gene 1 | -4.02 |
| Krtap7-1 | keratin associated protein 7-1 | -4.00 |
| Gje1 | gap junction protein, epsilon 1 | -3.95 |
| Hp | haptoglobin | -3.94 |
| Nos2 | nitric oxide synthase 2, inducible | -3.93 |
| Ccl20 | chemokine (C-C motif) ligand 20 | -3.89 |
| Ralgps2 | Ral GEF with PH domain and SH3 binding motif 2 | -3.80 |
| Ccdc19 | coiled-coil domain containing 19 | -3.75 |
| Afp | alpha fetoprotein | -3.70 |
| 4931429I11Rik | RIKEN cDNA 4931429I11 gene | -3.56 |
| Tmem90b | transmembrane protein 90B | -3.45 |
| Vnn3 | vanin 3 | -3.45 |
| Mageb16 | melanoma antigen family B, 16 | -3.15 |
| Scn2b | sodium channel, voltage-gated, type II, beta | -3.11 |
| Scand1 | SCAN domain-containing 1 | -3.11 |
| Esm1 | endothelial cell-specific molecule 1 | -3.06 |
| Adamts2 | a disintegrin-like and metallopeptidase (reprolysin type) with thrombospondin type 1 motif, 2 | -3.05 |
| Olfr1143 | olfactory receptor 1143 | -3.05 |
| 4930525F21Rik | RIKEN cDNA 4930525F21 gene | -3.05 |
| Olfr121 | olfactory receptor 121 | -3.05 |
| Lyn  \|LOC100862590 | Yamaguchi sarcoma viral (v-yes-1) oncogene homolog\|tyrosine-protein kinase Lyn-like | -3.05 |
| Amy1  \|Amy2a5  \|Amy2b | amylase 1, salivary\|amylase 2a5\|amylase 2b | -3.05 |
| Cyp27b1 | cytochrome P450, family 27, subfamily b, polypeptide 1 | -3.02 |
| Il1a | interleukin 1 alpha | -2.98 |
| Pabpc1l | poly(A) binding protein, cytoplasmic 1-like | -2.98 |
| Cmpk2 | cytidine monophosphate (UMP-CMP) kinase 2, mitochondrial | -2.96 |
| Adra1d | adrenergic receptor, alpha 1d | -2.93 |
| Gm10865 | predicted gene 10865 | -2.91 |
| Acsbg1 | acyl-CoA synthetase bubblegum family member 1 | -2.91 |
| Mcpt8 | mast cell protease 8 | -2.83 |
| Padi6 | peptidyl arginine deiminase, type VI | -2.82 |
| Kif21b | kinesin family member 21B | -2.80 |
| P2rx3 | purinergic receptor P2X, ligand-gated ion channel, 3 | -2.79 |
| Cyp39a1 | cytochrome P450, family 39, subfamily a, polypeptide 1 | -2.77 |
| Jph3 | junctophilin 3 | -2.76 |
| Il24 | interleukin 24 | -2.69 |
| Gm4070  \|Gvin1  \|Gm8989  \|Gm8979 | predicted gene 4070\|GTPase, very large interferon inducible 1\|very large inducible GTPase 1 pseudogene\|very large inducible GTPase 1 pseudogene | -2.68 |
| C3 | complement component 3 | -2.67 |
| Pom121l2 | POM121 membrane glycoprotein-like 2 (rat) | -2.66 |
| Lgi2 | leucine-rich repeat LGI family, member 2 | -2.66 |
| Igfl3 | IGF-like family member 3 | -2.65 |
| Pigz | phosphatidylinositol glycan anchor biosynthesis, class Z | -2.63 |
| H2-Aa | histocompatibility 2, class II antigen A, alpha | -2.61 |
| Dock8 | dedicator of cytokinesis 8 | -2.55 |
| Bst1 | bone marrow stromal cell antigen 1 | -2.50 |
| Lama3 | laminin, alpha 3 | -2.47 |
| Srgn | serglycin | -2.47 |
| Mettl7b | methyltransferase like 7B | -2.45 |
| Ubqln2 | ubiquilin 2 | -2.43 |
| Cass4 | Cas scaffolding protein family member 4 | -2.41 |
| Psmb9 | proteasome (prosome, macropain) subunit, beta type 9 (large multifunctional peptidase 2) | -2.40 |
| Fam189a2 | family with sequence similarity 189, member A2 | -2.40 |
| Yipf7 | Yip1 domain family, member 7 | -2.39 |
| Slc12a5 | solute carrier family 12, member 5 | -2.39 |
| Fam169b | family with sequence similarity 169, member B | -2.33 |
| Slc44a3 | solute carrier family 44, member 3 | -2.31 |
| Cxcl5 | chemokine (C-X-C motif) ligand 5 | -2.31 |
| Ifi47 | interferon gamma inducible protein 47 | -2.30 |
| Angptl7 | angiopoietin-like 7 | -2.28 |
| Vmn1r66 | vomeronasal 1 receptor 66 | -2.25 |
| Dcn | decorin | -2.24 |
| Fyb | FYN binding protein | -2.20 |
| Foxn1 | forkhead box N1 | -2.20 |
| Snord71 | small nucleolar RNA, C/D box 71 | -2.20 |
| Yaf2 | YY1 associated factor 2 | -2.19 |
| Dcn | decorin | -2.18 |
| P2ry6 | pyrimidinergic receptor P2Y, G-protein coupled, 6 | -2.17 |
| Glipr2 | GLI pathogenesis-related 2 | -2.16 |
| Niacr1 | niacin receptor 1 | -2.16 |
| Slc12a1 | solute carrier family 12, member 1 | -2.16 |
| Abcc3 | ATP-binding cassette, sub-family C (CFTR/MRP), member 3 | -2.13 |
| Syt2 | synaptotagmin II | -2.10 |
| Usp18 | ubiquitin specific peptidase 18 | -2.08 |
| Irf1 | interferon regulatory factor 1 | -2.08 |
| Oasl1 | 2'-5' oligoadenylate synthetase-like 1 | -2.08 |
| Pla1a | phospholipase A1 member A | -2.08 |
| Foxo3 | forkhead box O3 | -2.07 |
| Sh2b2 | SH2B adaptor protein 2 | -2.06 |
| Slc39a4 | solute carrier family 39 (zinc transporter), member 4 | -2.06 |
| Ccl7 | chemokine (C-C motif) ligand 7 | -2.03 |
| Khdrbs2 | KH domain containing, RNA binding, signal transduction associated 2 | -2.01 |
| Ahsa2 | AHA1, activator of heat shock protein ATPase homolog 2 (yeast) | -2.00 |
| Avil | advillin | -2.00 |
| Prox2 | prospero homeobox 2 | -1.99 |
| Scarna6 | small Cajal body-specific RNA 6 | -1.98 |
| Spata13 | spermatogenesis associated 13 | -1.98 |
| Unc13a | unc-13 homolog A (C. elegans) | -1.97 |
| Timp3 | tissue inhibitor of metalloproteinase 3 | -1.97 |
| Vip | vasoactive intestinal polypeptide | -1.96 |
| Olfr564 | olfactory receptor 564 | -1.95 |
| Clec4a3 | C-type lectin domain family 4, member a3 | -1.93 |
| Car6 | carbonic anhydrase 6 | -1.93 |
| Amelx | amelogenin X chromosome | -1.91 |
| Cxcl2 | chemokine (C-X-C motif) ligand 2 | -1.90 |
| C1ra\|C1rb | complement component 1, r subcomponent A\|complement component 1, r subcomponent B | -1.89 |
| Muc15 | mucin 15 | -1.88 |
| Tap1 | transporter 1, ATP-binding cassette, sub-family B (MDR/TAP) | -1.86 |
| P4ha2 | procollagen-proline, 2-oxoglutarate 4-dioxygenase (proline 4-hydroxylase), alpha II polypeptide | -1.85 |
| Mmp10 | matrix metallopeptidase 10 | -1.85 |
| Cnga1 | cyclic nucleotide gated channel alpha 1 | -1.83 |
| Ehf | ets homologous factor | -1.83 |
| Lcn2 | lipocalin 2 | -1.82 |
| Cldn3 | claudin 3 | -1.81 |
| Arrdc4 | arrestin domain containing 4 | -1.81 |
| Csf3 | colony stimulating factor 3 (granulocyte) | -1.79 |
| Olfr1 | olfactory receptor 1 | -1.79 |
| Prtn3 | proteinase 3 | -1.78 |
| Irg1 | immunoresponsive gene 1 | -1.77 |
| Baalc | brain and acute leukemia, cytoplasmic | -1.77 |
| Adamts8 | a disintegrin-like and metallopeptidase (reprolysin type) with thrombospondin type 1 motif, 8 | -1.77 |
| Lass3 | LAG1 homolog, ceramide synthase 3 | -1.75 |
| Il23a | interleukin 23, alpha subunit p19 | -1.75 |
| Il6 | interleukin 6 | -1.75 |
| Cblc | Casitas B-lineage lymphoma c | -1.75 |
| Lrrc8e | leucine rich repeat containing 8 family, member E | -1.74 |
| Ccl3 | chemokine (C-C motif) ligand 3 | -1.73 |
| Agphd1 | aminoglycoside phosphotransferase domain containing 1 | -1.73 |
| Chrm1 | cholinergic receptor, muscarinic 1, CNS | -1.73 |
| Angptl4 | angiopoietin-like 4 | -1.73 |
| Cd8a | CD8 antigen, alpha chain | -1.73 |
| Il22ra1 | interleukin 22 receptor, alpha 1 | -1.72 |
| Muc20 | mucin 20 | -1.71 |
| Ms4a10 | membrane-spanning 4-domains, subfamily A, member 10 | -1.71 |
| Rcvrn | recoverin | -1.71 |
| Ddx60 | DEAD (Asp-Glu-Ala-Asp) box polypeptide 60 | -1.71 |
| Arrdc4 | arrestin domain containing 4 | -1.70 |
| Phkg1 | phosphorylase kinase gamma 1 | -1.70 |
| Apol10b | apolipoprotein L 10b | -1.69 |
| Psmb8 | proteasome (prosome, macropain) subunit, beta type 8 (large multifunctional peptidase 7) | -1.69 |
| Naip2 | NLR family, apoptosis inhibitory protein 2 | -1.68 |
| Abcb1a | ATP-binding cassette, sub-family B (MDR/TAP), member 1A | -1.67 |
| Gpr12 | G-protein coupled receptor 12 | -1.67 |
| Tmco5 | transmembrane and coiled-coil domains 5 | -1.66 |
| Zfp36l3 | zinc finger protein 36, C3H type-like 3 | -1.66 |
| Olfr52 | olfactory receptor 52 | -1.66 |
| Rnf122 | ring finger protein 122 | -1.66 |
| Spnb5 | spectrin beta 5 | -1.66 |
| Lhx9 | LIM homeobox protein 9 | -1.66 |
| Dapk2 | death-associated protein kinase 2 | -1.66 |
| Gbp1 | guanylate binding protein 1 | -1.64 |
| Prelp | proline arginine-rich end leucine-rich repeat | -1.64 |
| Sept4 | septin 4 | -1.64 |
| Rtp4 | receptor transporter protein 4 | -1.63 |
| Olfr1449 | olfactory receptor 1449 | -1.63 |
| Csf2 | colony stimulating factor 2 (granulocyte-macrophage) | -1.62 |
| Gbp2 | guanylate binding protein 2 | -1.62 |
| Clec2d | C-type lectin domain family 2, member d | -1.61 |
| Gpm6b | glycoprotein m6b | -1.61 |
| Olfr610 | olfactory receptor 610 | -1.60 |
| Slc4a4 | solute carrier family 4 (anion exchanger), member 4 | -1.59 |
| H2-Ab1 | histocompatibility 2, class II antigen A, beta 1 | -1.59 |
| Enpp1 | ectonucleotide pyrophosphatase/phosphodiesterase 1 | -1.59 |
| Cd68 | CD68 antigen | -1.59 |
| Zim1 | zinc finger, imprinted 1 | -1.58 |
| Fbxw18 | F-box and WD-40 domain protein 18 | -1.58 |
| Slc30a10 | solute carrier family 30, member 10 | -1.58 |
| Nrip2 | nuclear receptor interacting protein 2 | -1.58 |
| Bnc2 | basonuclin 2 | -1.56 |
| Reep2 | receptor accessory protein 2 | -1.56 |
| Bst2 | bone marrow stromal cell antigen 2 | -1.55 |
| Acpp | acid phosphatase, prostate | -1.54 |
| Apol9a  \|Apol9b | apolipoprotein L 9a\|apolipoprotein L 9b | -1.54 |
| Dpy30 | dpy-30 homolog (C. elegans) | -1.54 |
| Mmp19 | matrix metallopeptidase 19 | -1.52 |
| Nt5e | 5' nucleotidase, ecto | -1.52 |
| Itga2 | integrin alpha 2 | -1.52 |
| Dennd2d | DENN/MADD domain containing 2D | -1.51 |
| Gng2 | guanine nucleotide binding protein (G protein), gamma 2 | -1.51 |
| Hhip | Hedgehog-interacting protein | -1.51 |
| Pax9 | paired box gene 9 | -1.51 |
| Lilrb4 | leukocyte immunoglobulin-like receptor, subfamily B, member 4 | -1.51 |
| Cpm | carboxypeptidase M | -1.51 |
