# Supplementary material for: TGF‐β1 secreted by Tregs in lymph nodes promotes breast cancer malignancy via up‐regulation of IL‐17RB
Source: EMBO Mol Med. 2017 Oct 9;9(12):1660–80. doi: 10.15252/emmm.201606914 (PMC5709760; doi:10.15252/emmm.201606914)

Figure 3C
Boxes highlight lanes used in the figure

Il-17rb

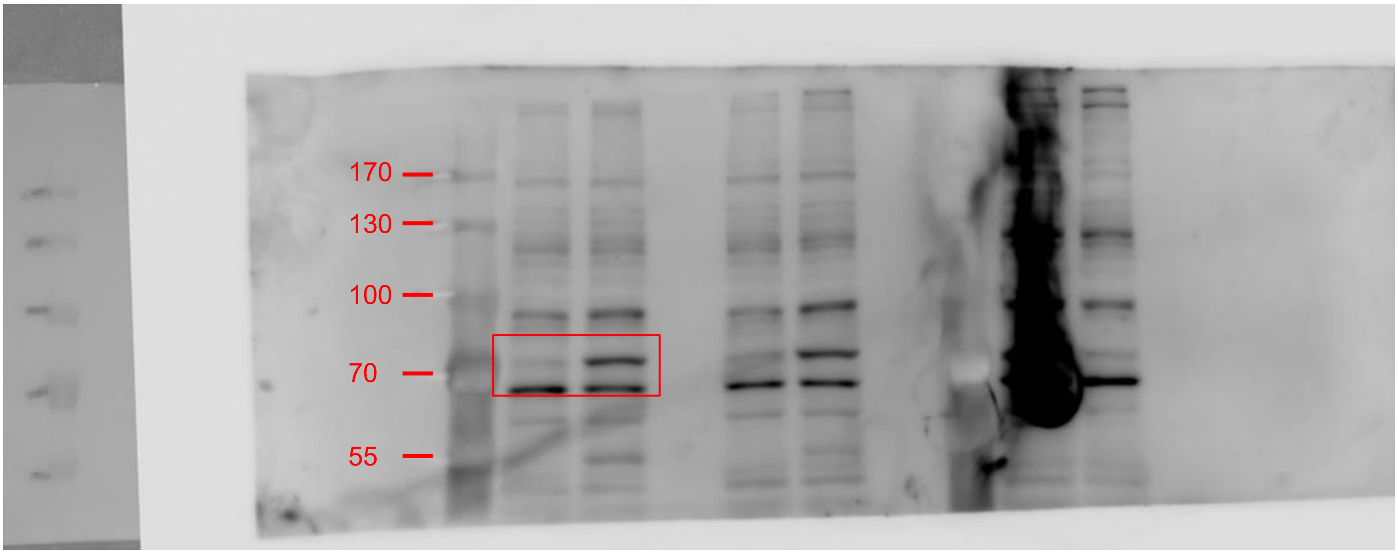

Gapdh

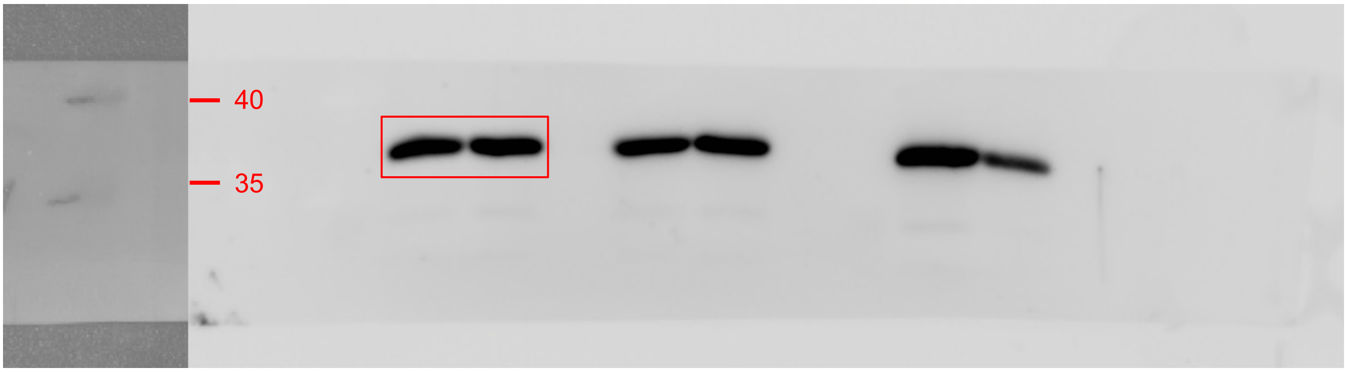

Figure 3D
Boxes highlight lanes used in the figure

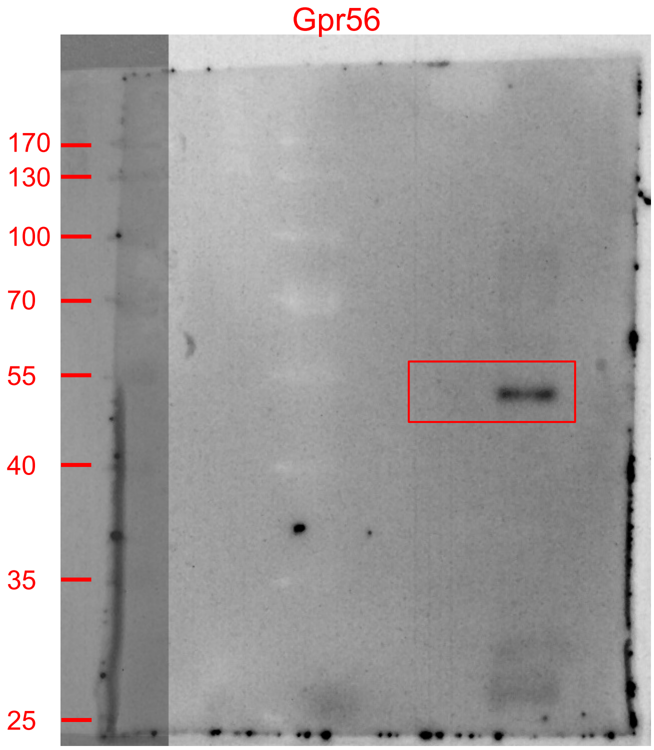

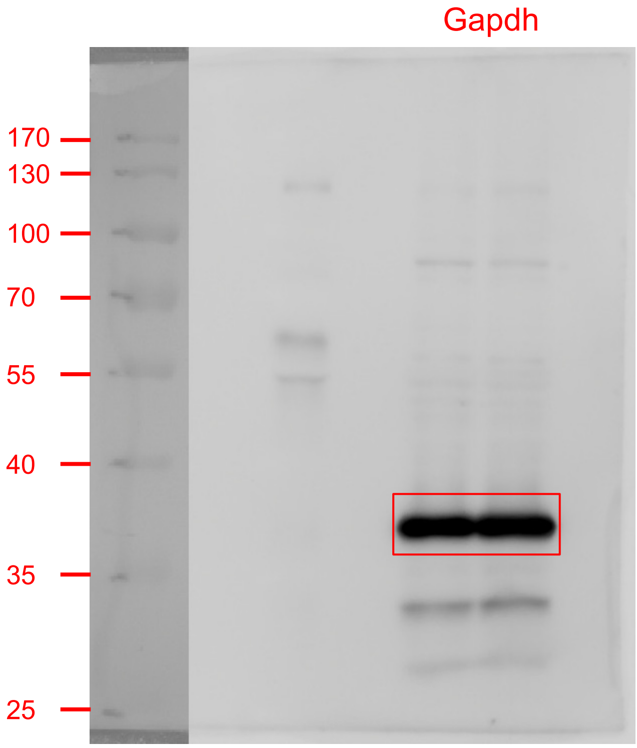

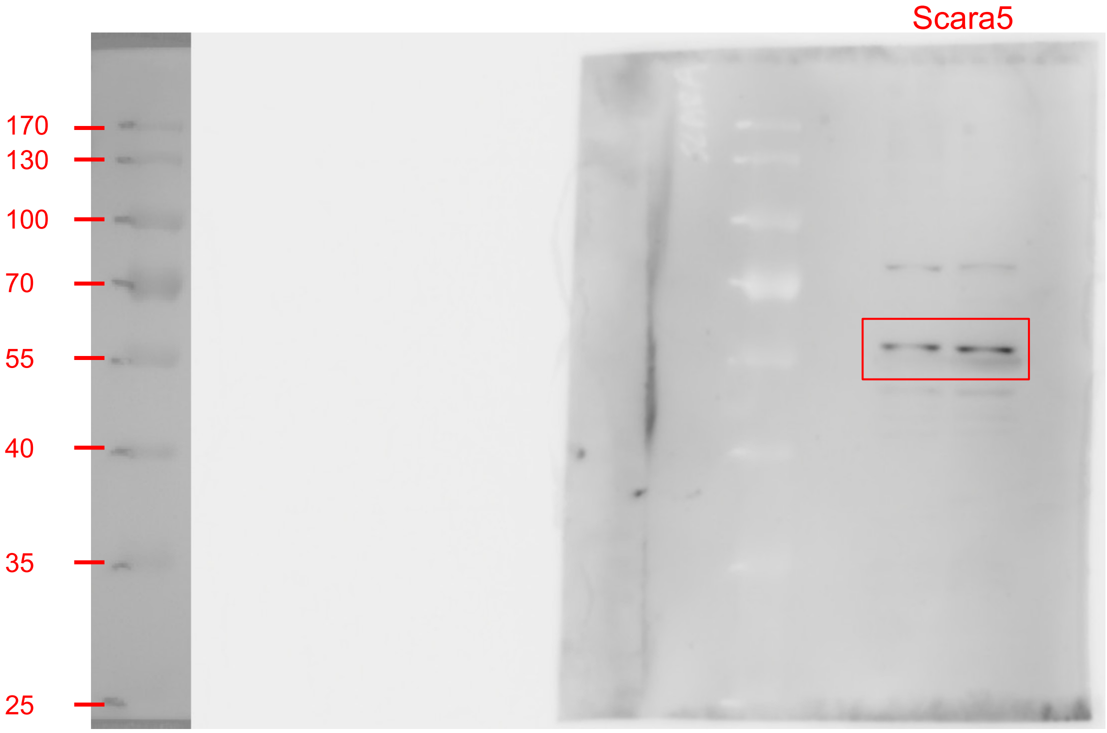

Figure 3E
Boxes highlight lanes used in the figure

Il-17rb

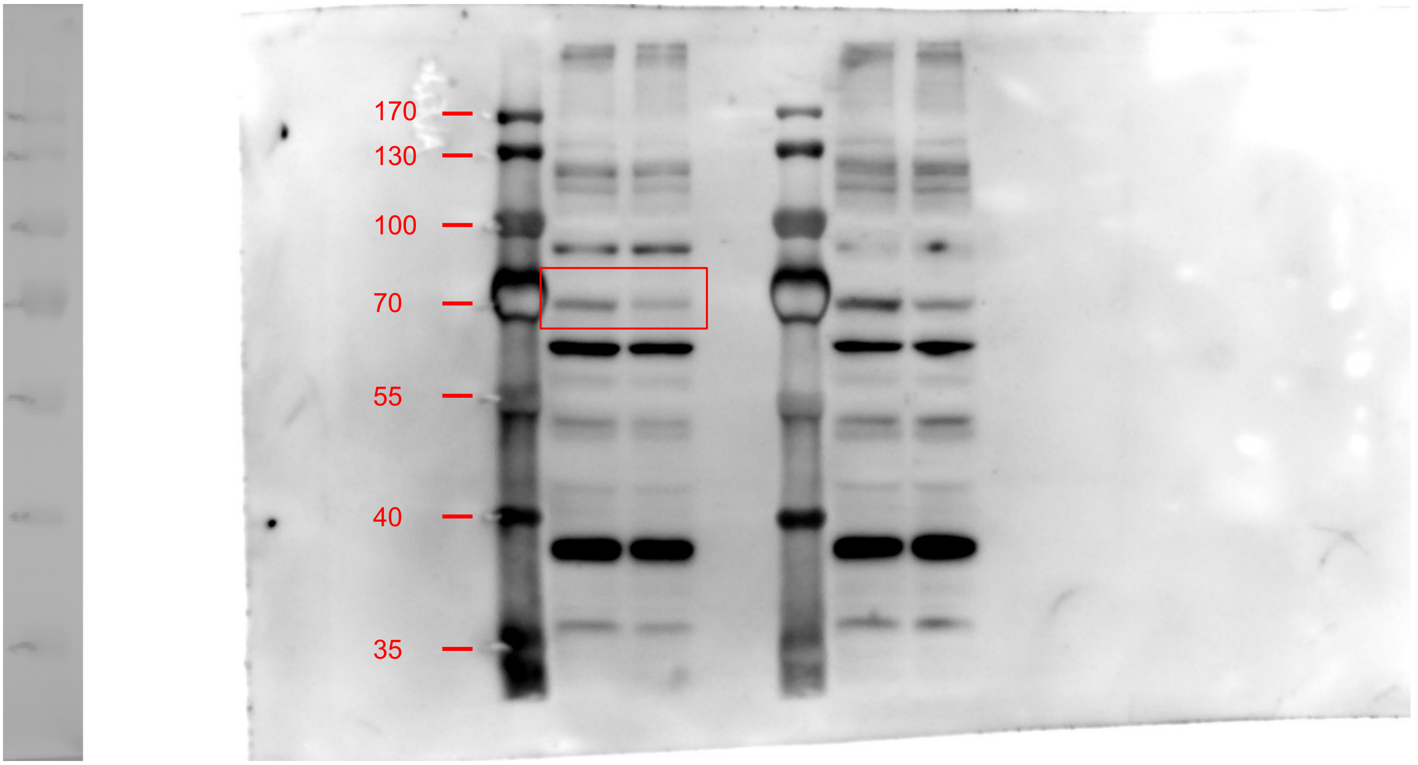

Gapdh

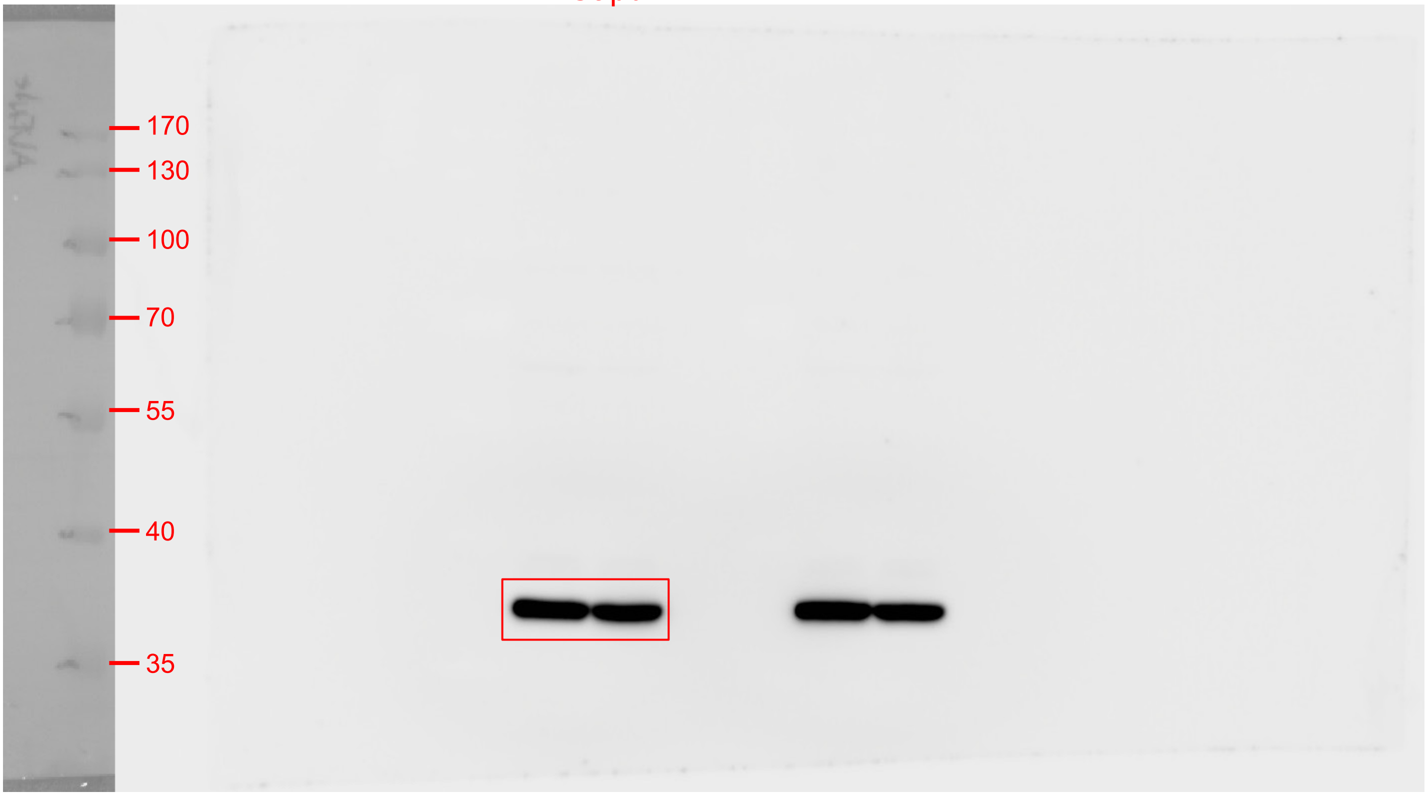

Figure 3E
Boxes highlight lanes used in the figure

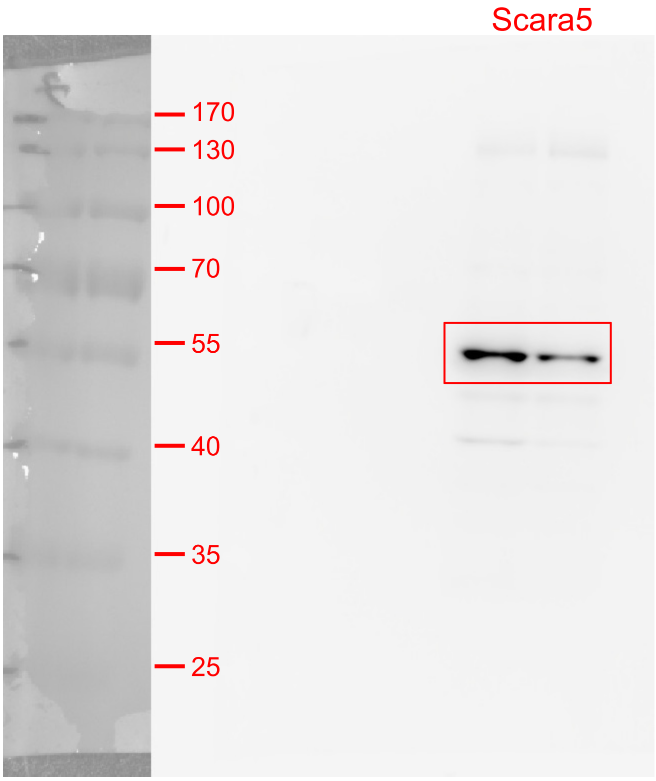

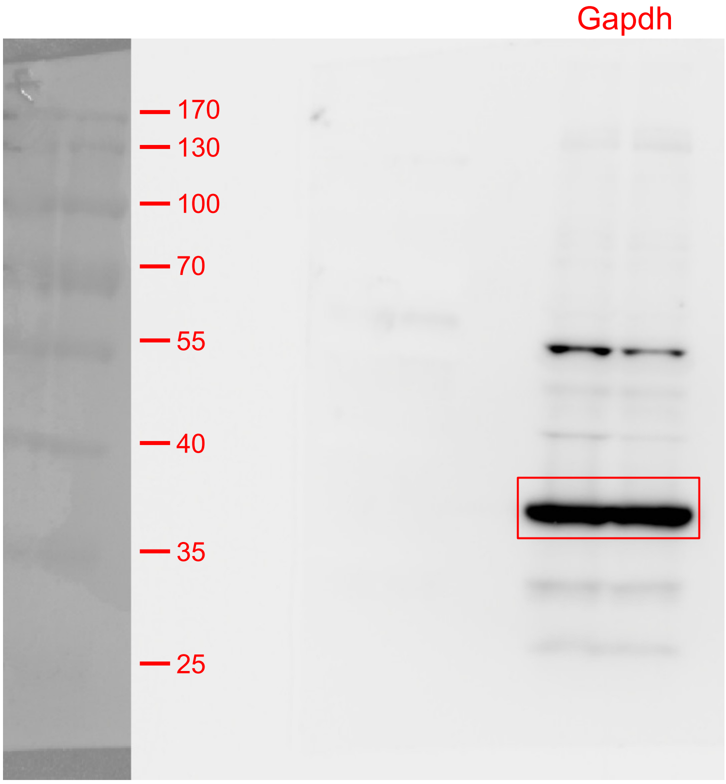

Figure 3G
Boxes highlight lanes used in the figure

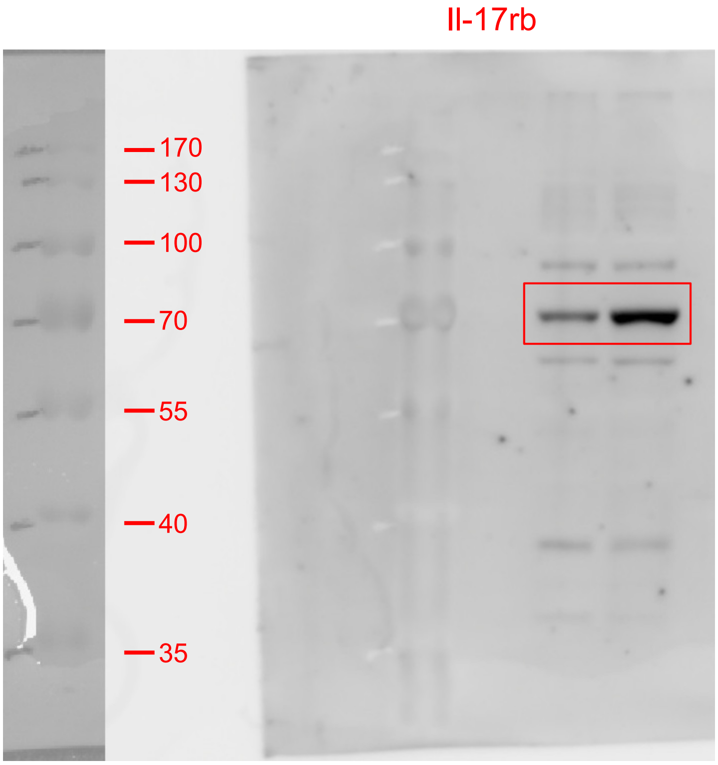

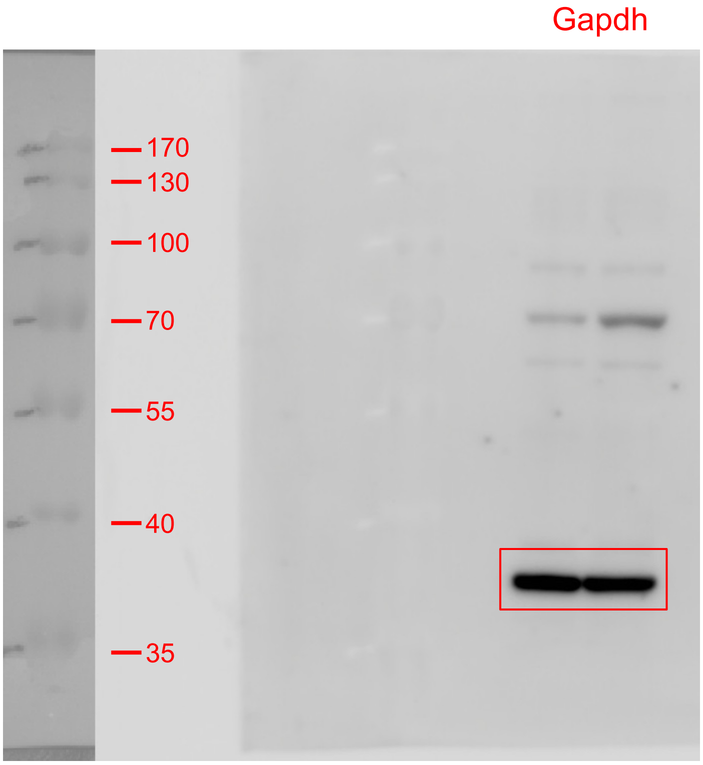

Figure 3I
Boxes highlight lanes used in the figure

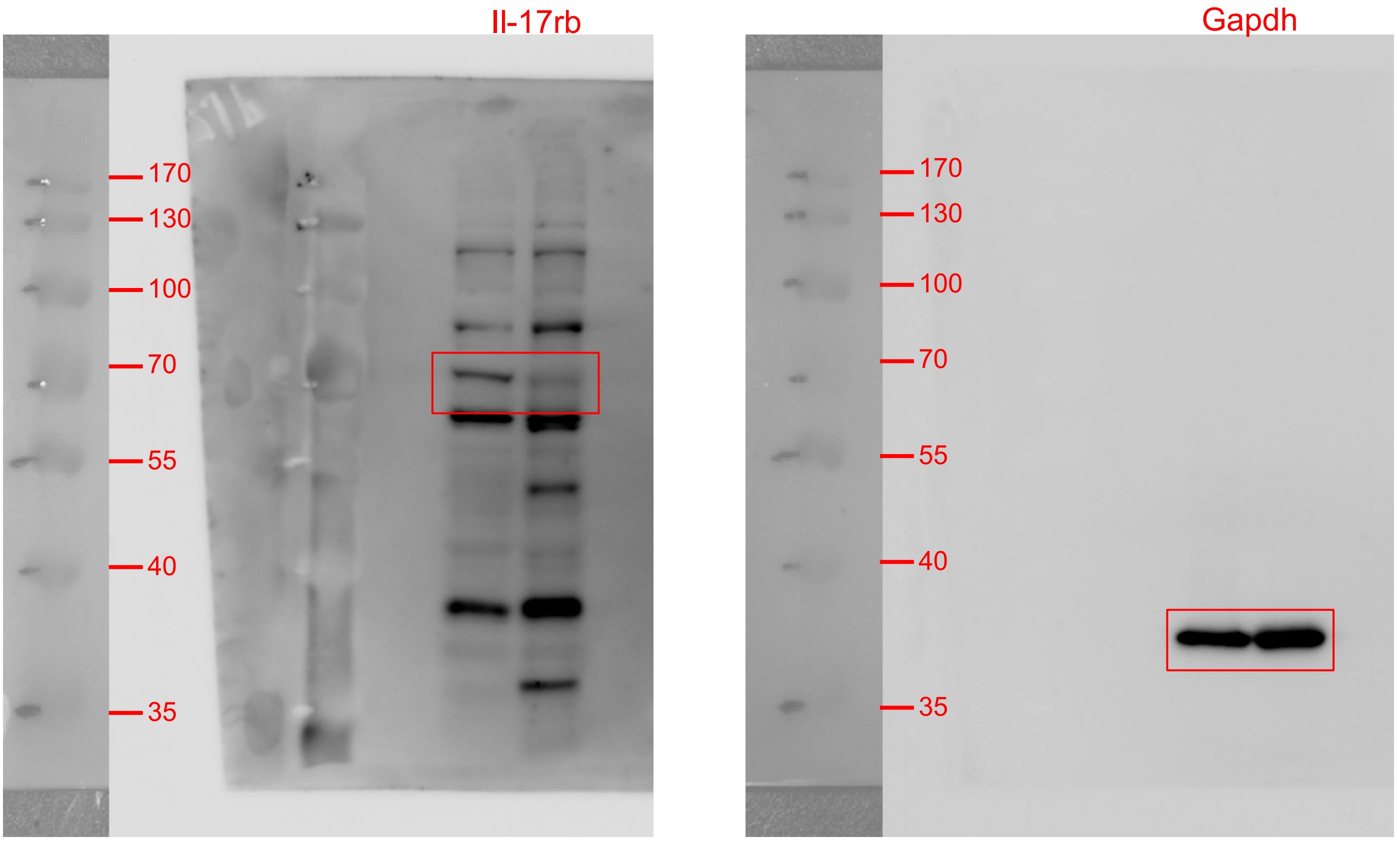

Supplement: Supplementary file 6 — Source Data for Figure 3 [file EMMM-9-1660-s005.pdf]
