# Supplementary material for: TGF‐β1 secreted by Tregs in lymph nodes promotes breast cancer malignancy via up‐regulation of IL‐17RB
Source: EMBO Mol Med. 2017 Oct 9;9(12):1660–80. doi: 10.15252/emmm.201606914 (PMC5709760; doi:10.15252/emmm.201606914)

Figure 4C
Boxes highlight lanes used in the figure

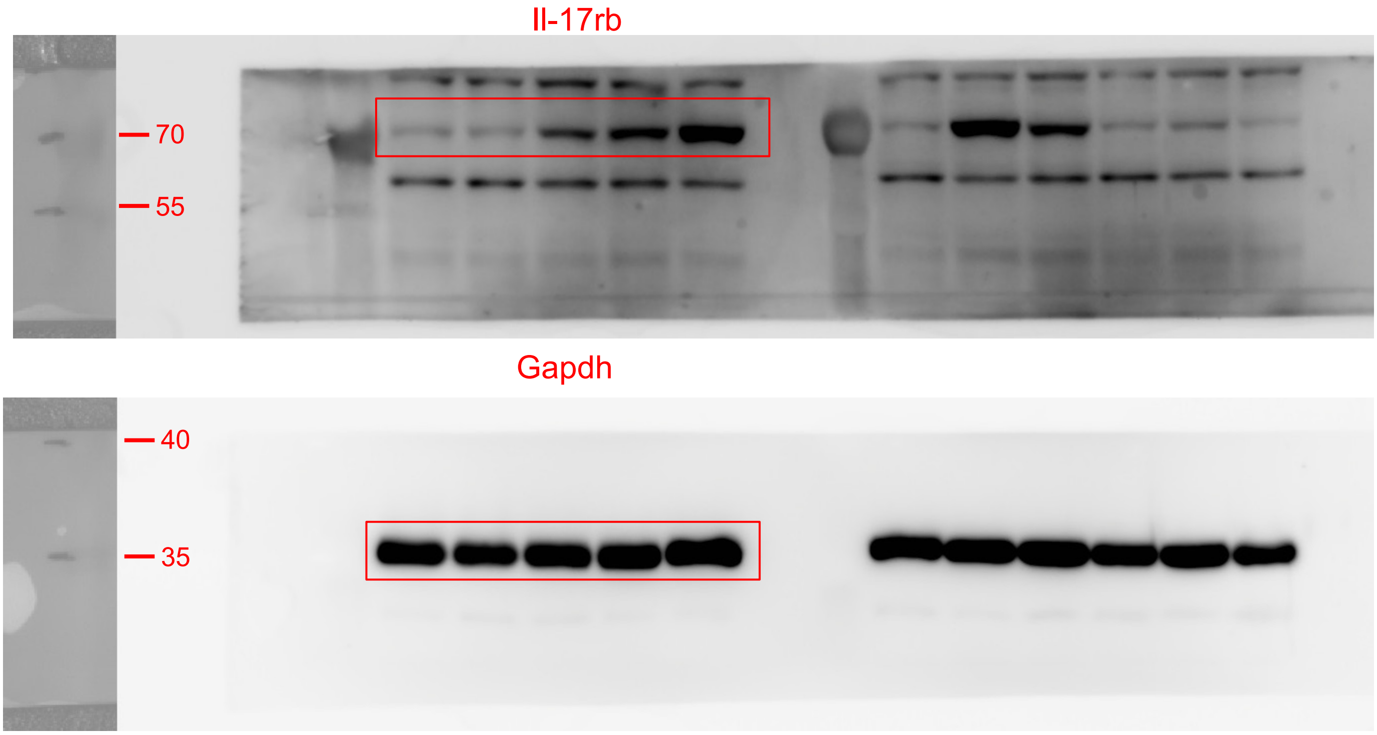

Figure 4F
Boxes highlight lanes used in the figure

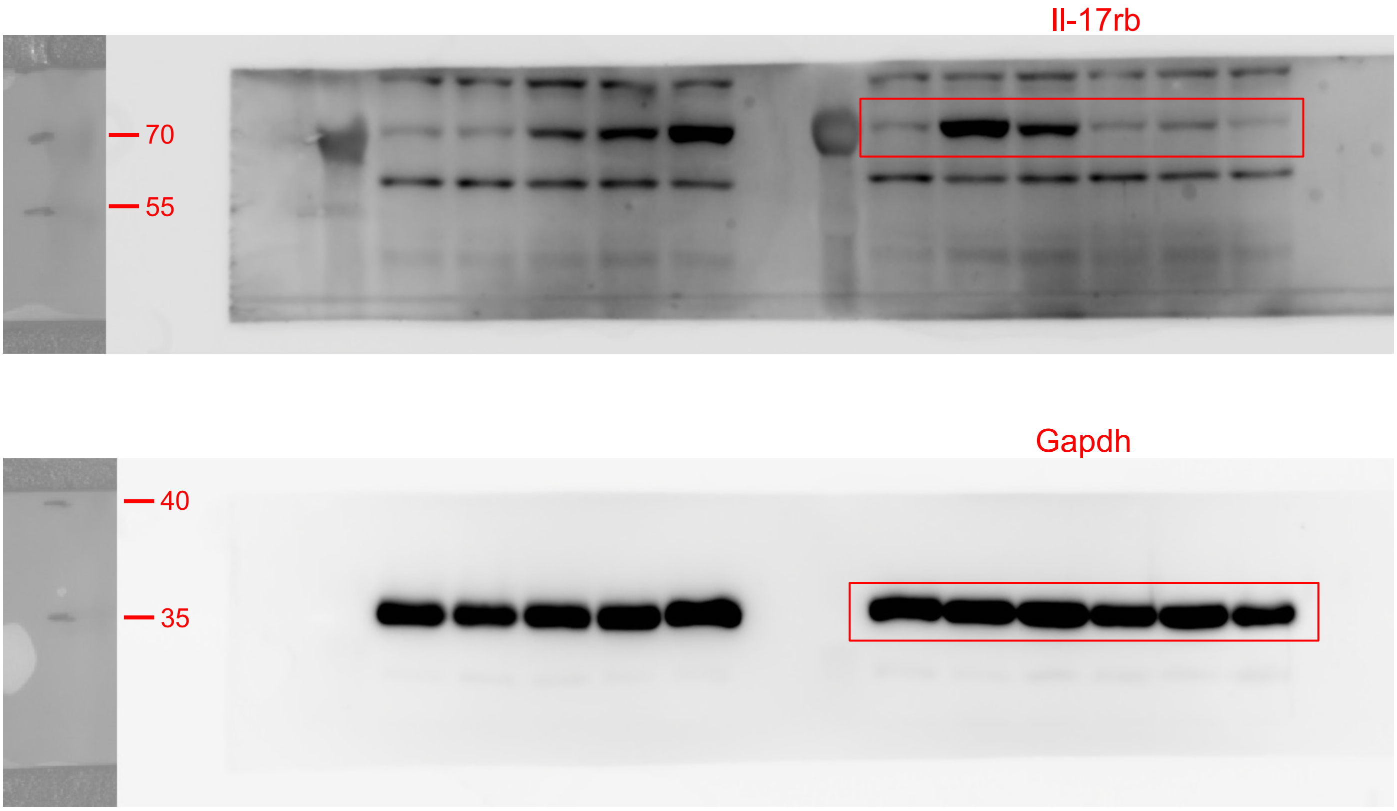

Supplement: Supplementary file 7 — Source Data for Figure 4 [file EMMM-9-1660-s006.pdf]
