# Supplementary figures and images for: TGF‐β1 secreted by Tregs in lymph nodes promotes breast cancer malignancy via up‐regulation of IL‐17RB
Source: EMBO Mol Med. 2017 Oct 9;9(12):1660–80. doi: 10.15252/emmm.201606914 (PMC5709760; doi:10.15252/emmm.201606914)

Figure 5B  
Boxes highlight lanes used in the figure

Il-17rb

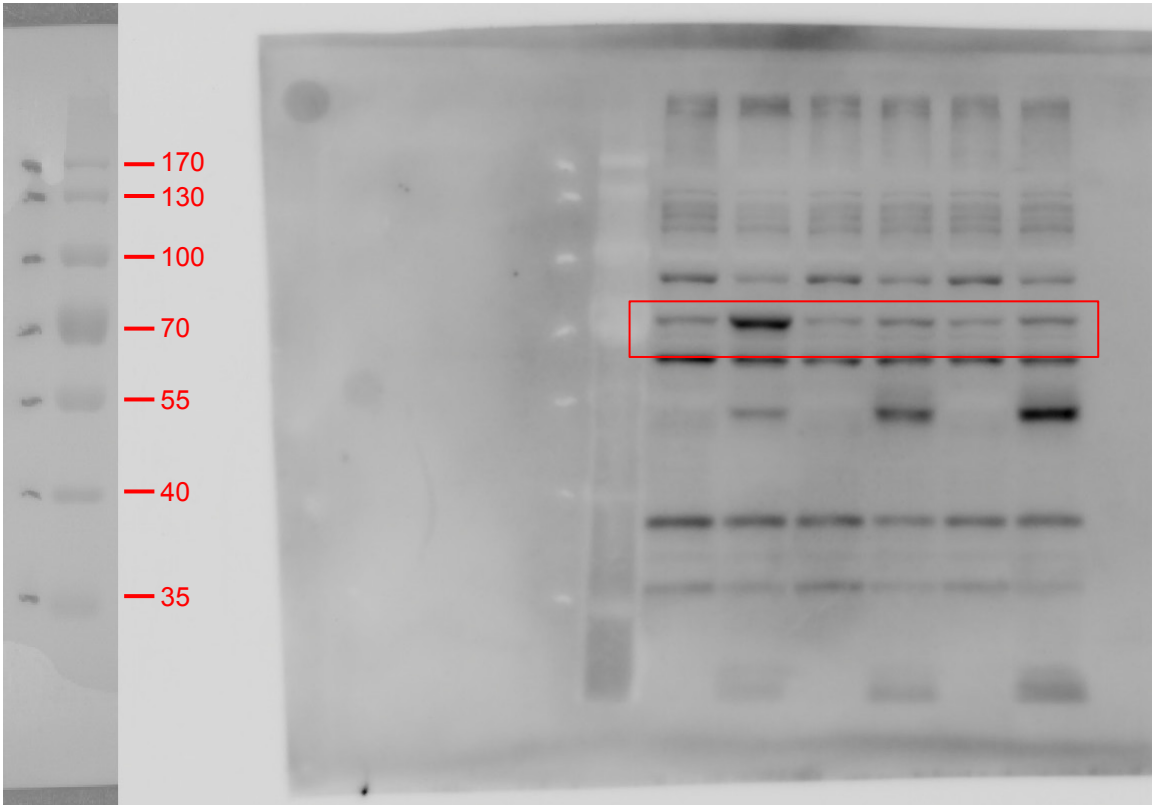

Gapdh

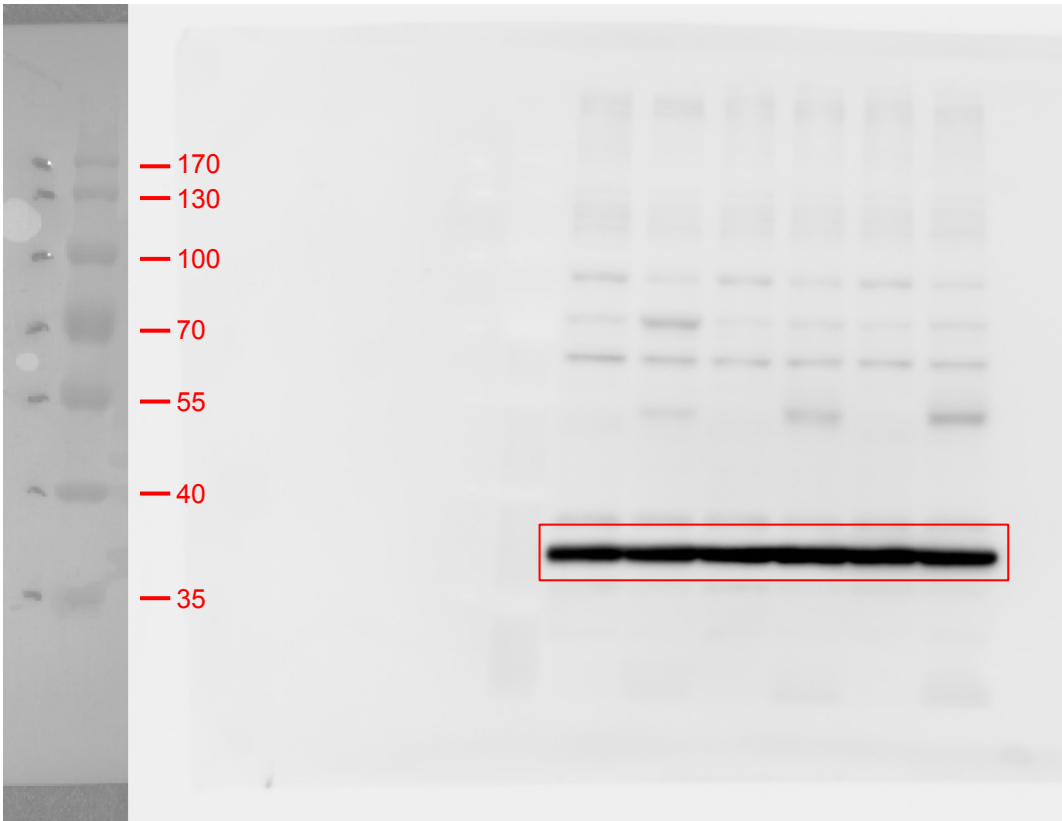

Supplement: Supplementary file 8 — Source Data for Figure 5 [file EMMM-9-1660-s007.pdf]

Appendix Figure S2  
Boxes highlight lanes used in the figure

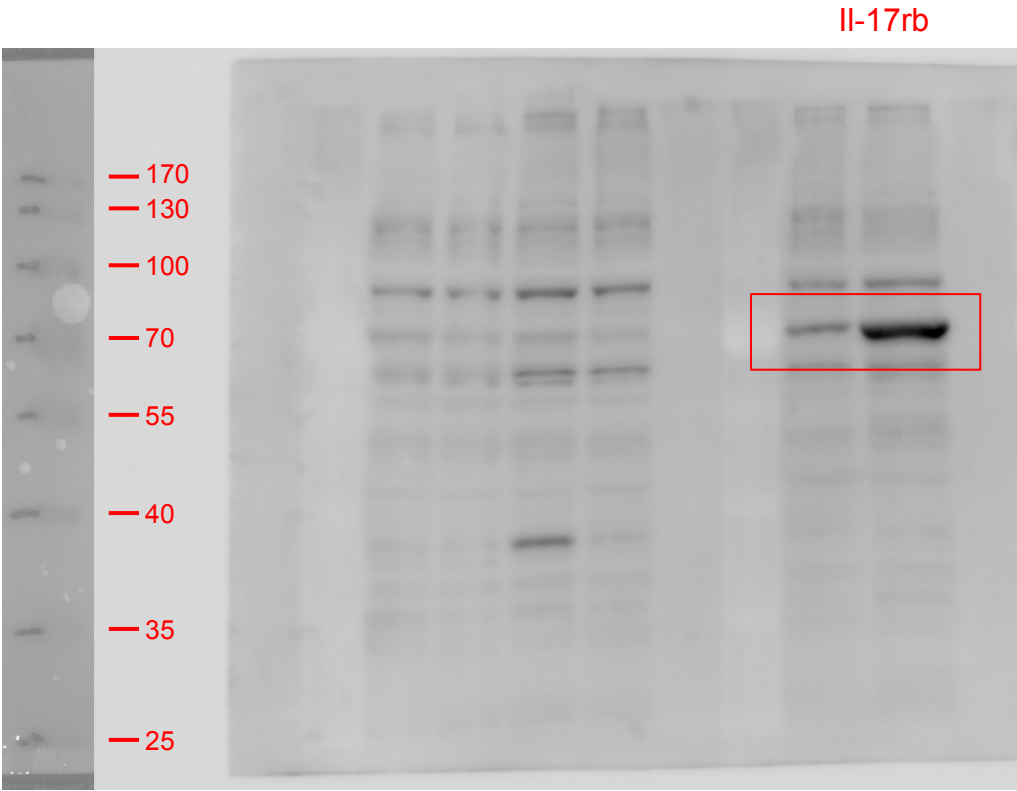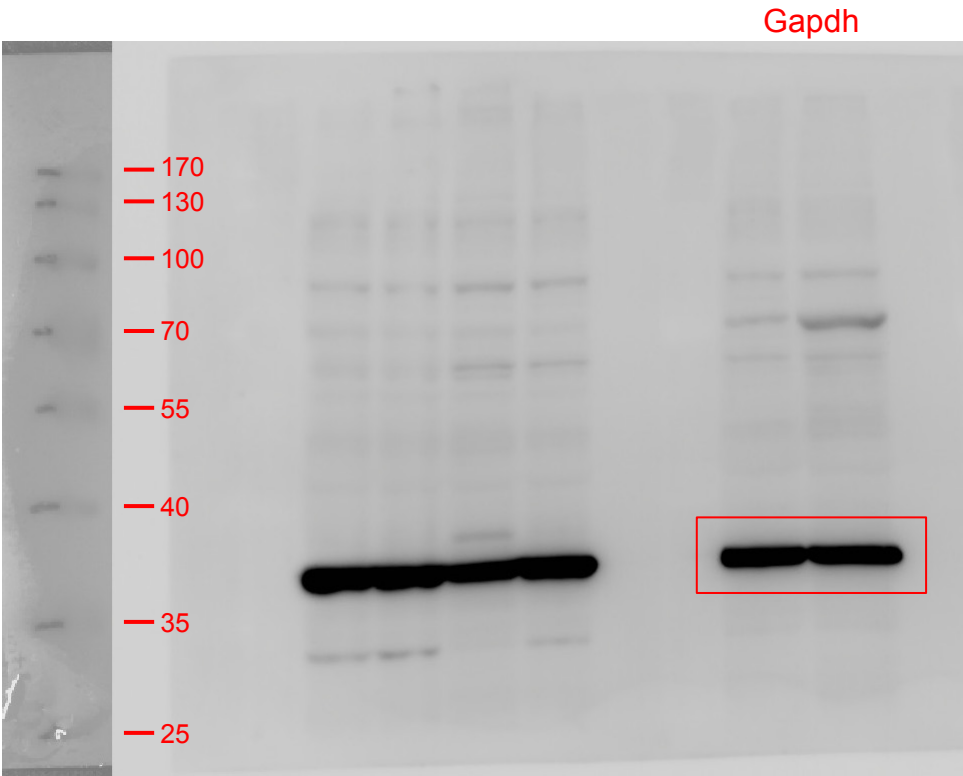

Supplement: Supplementary file 10 — Source Data for Expanded View and Appendix [file EMMM-9-1660-s009.zip › EMM06914source_dataEVandAppendix/EMM-06914-source_data/Fig_S2_source_data.pdf]
