# Supplementary material for: TGF‐β1 secreted by Tregs in lymph nodes promotes breast cancer malignancy via up‐regulation of IL‐17RB
Source: EMBO Mol Med. 2017 Oct 9;9(12):1660–80. doi: 10.15252/emmm.201606914 (PMC5709760; doi:10.15252/emmm.201606914)

Figure 6B

Boxes highlight lanes used in the figure

Il-17rb

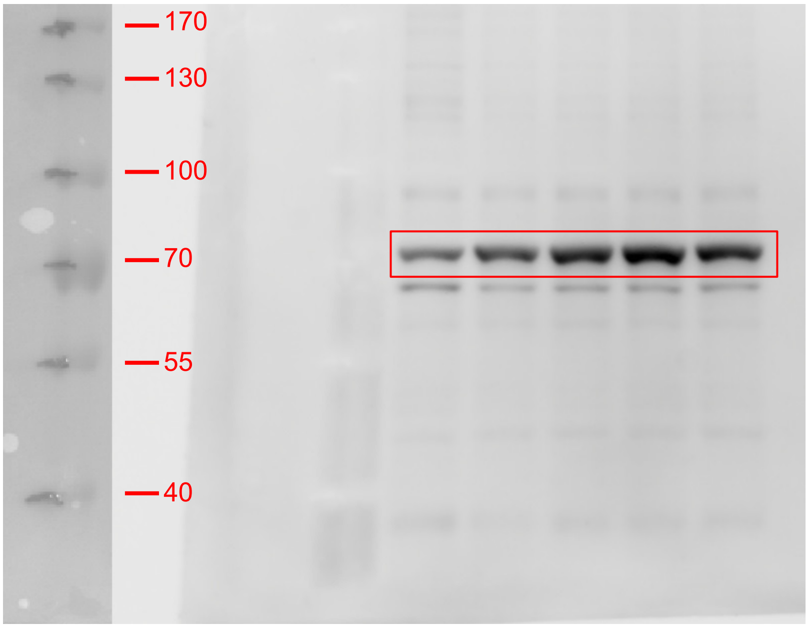

α-tubulin

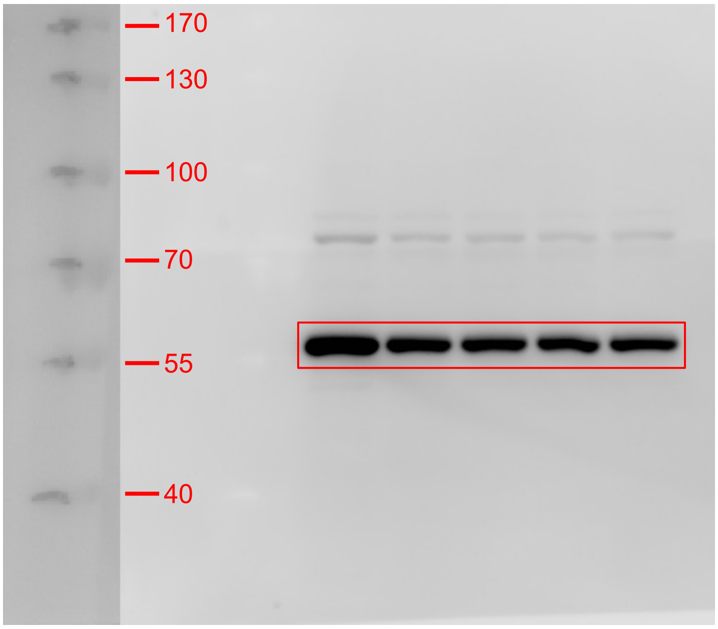

Figure 6C
Boxes highlight lanes used in the figure

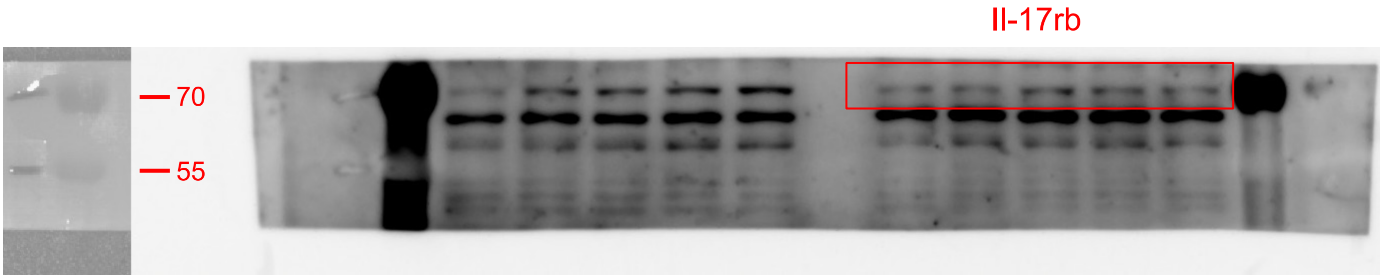

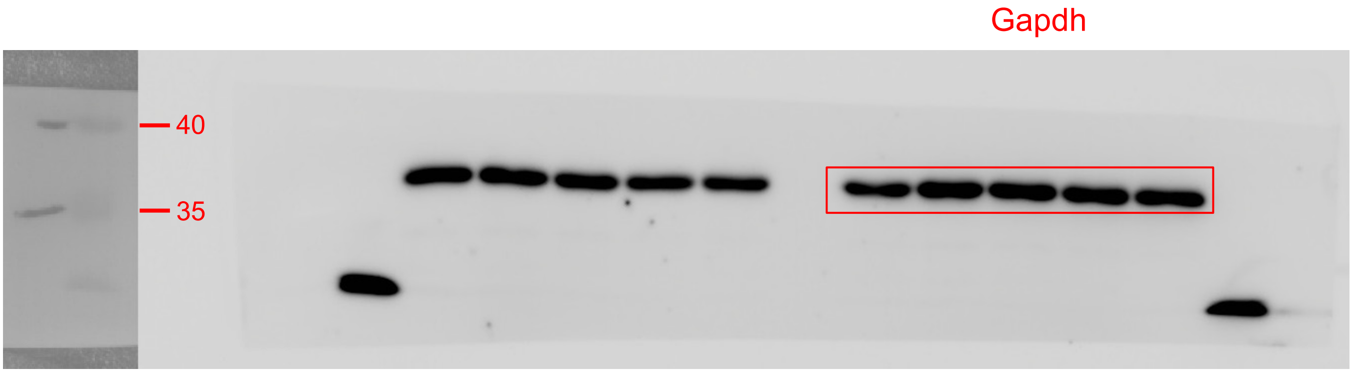

Figure 6D

Boxes highlight lanes used in the figure

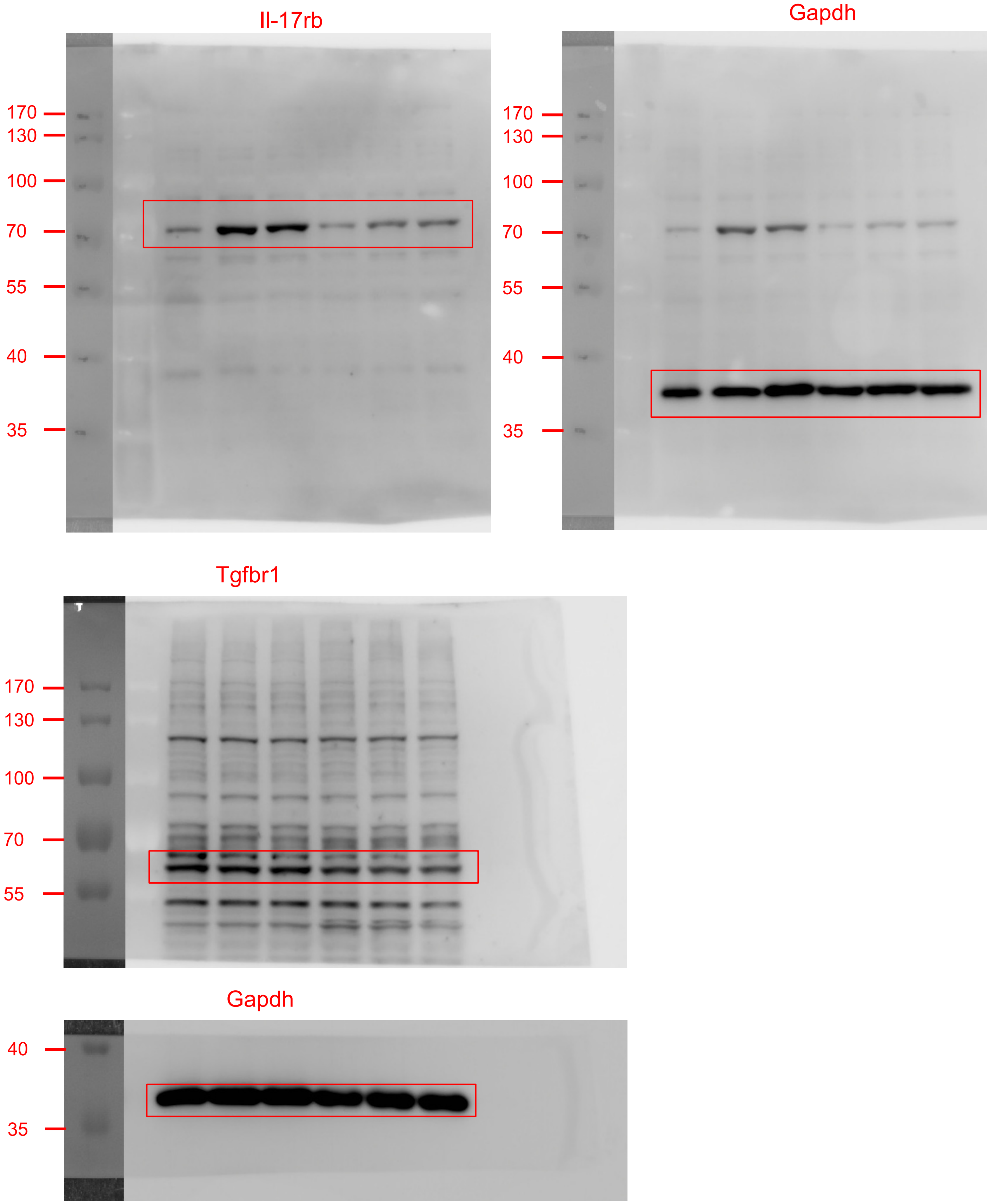

Figure 6F
Boxes highlight lanes used in the figure

Il-17rb

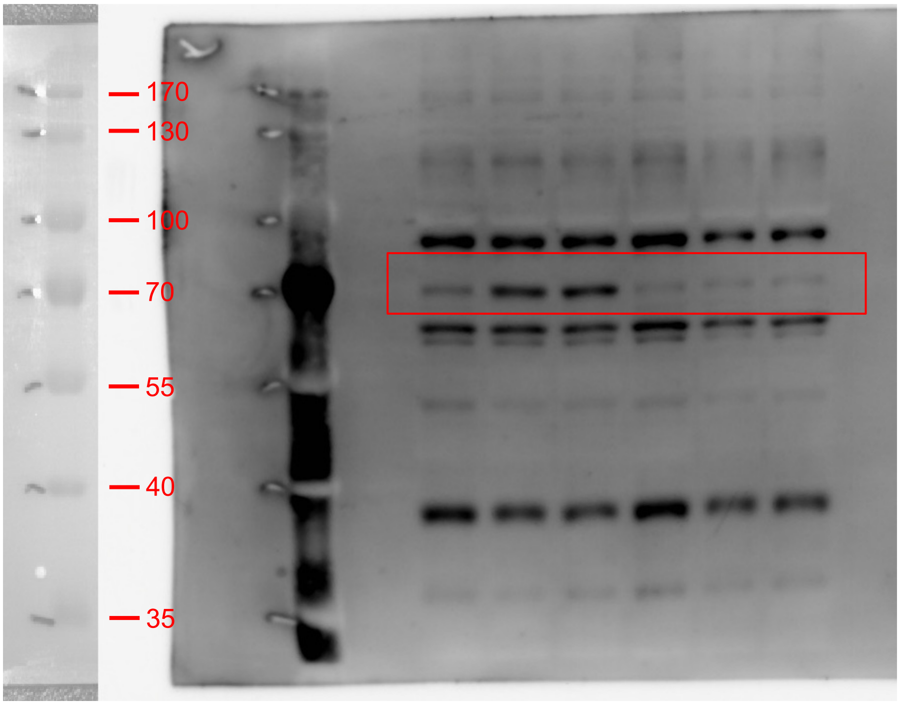

Smad2

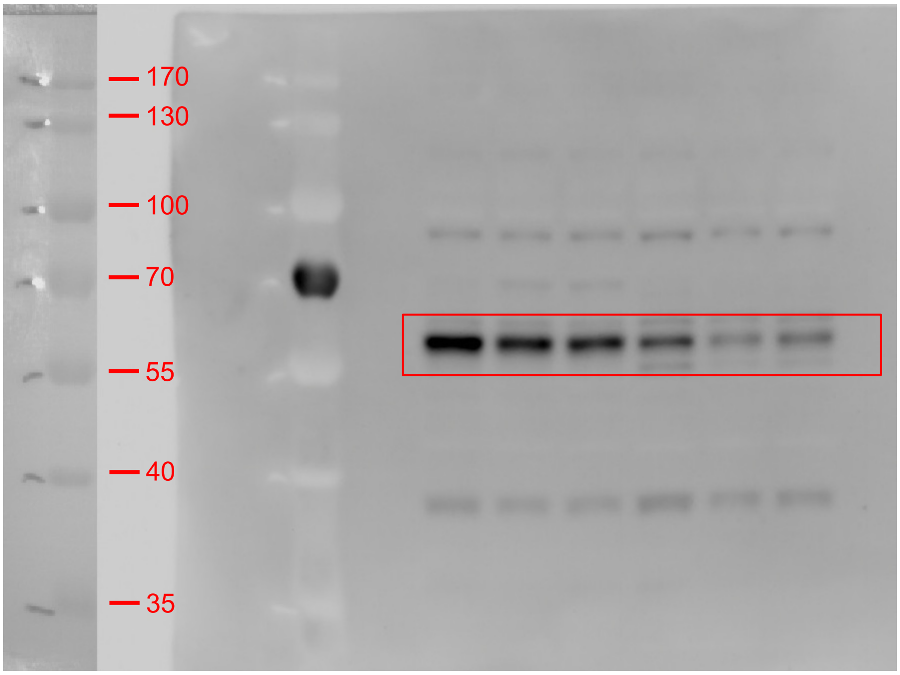

Figure 6F
Boxes highlight lanes used in the figure

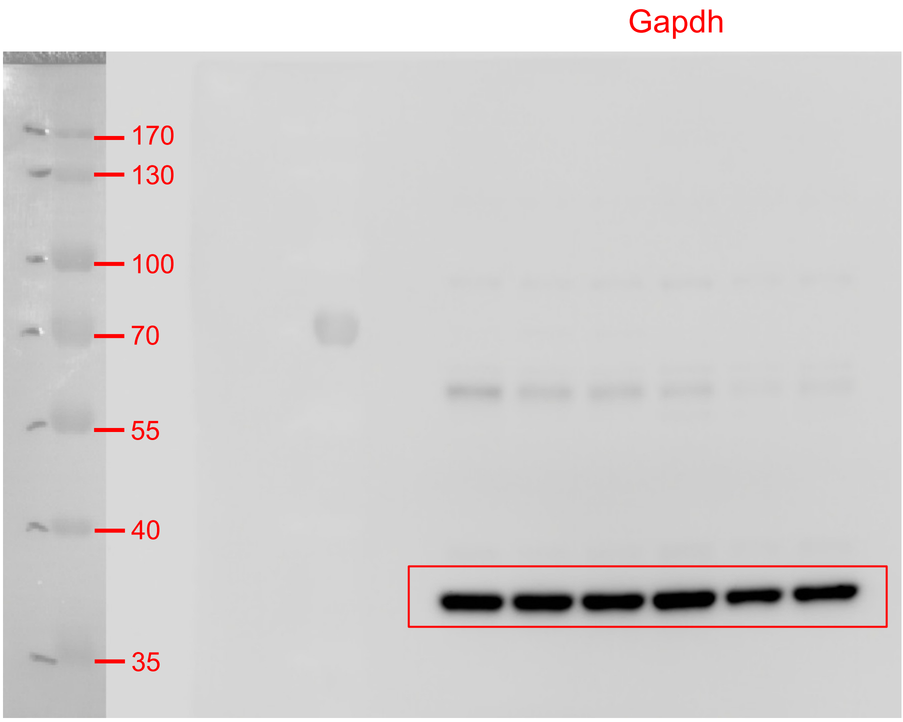

Figure 6G
Boxes highlight lanes used in the figure

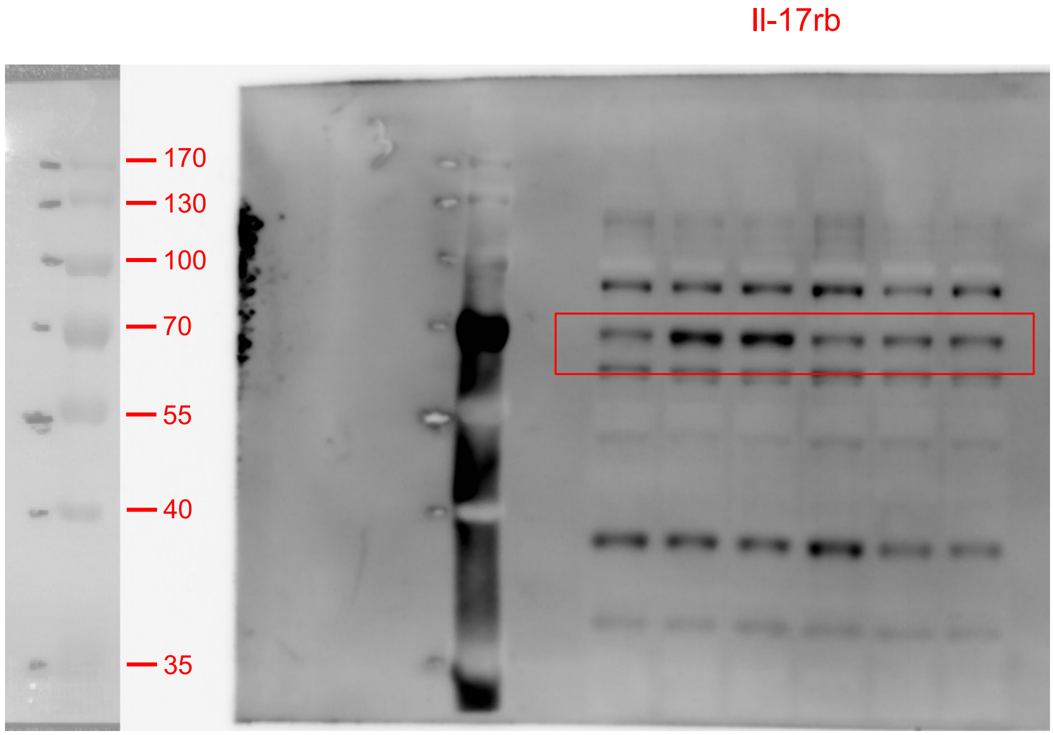

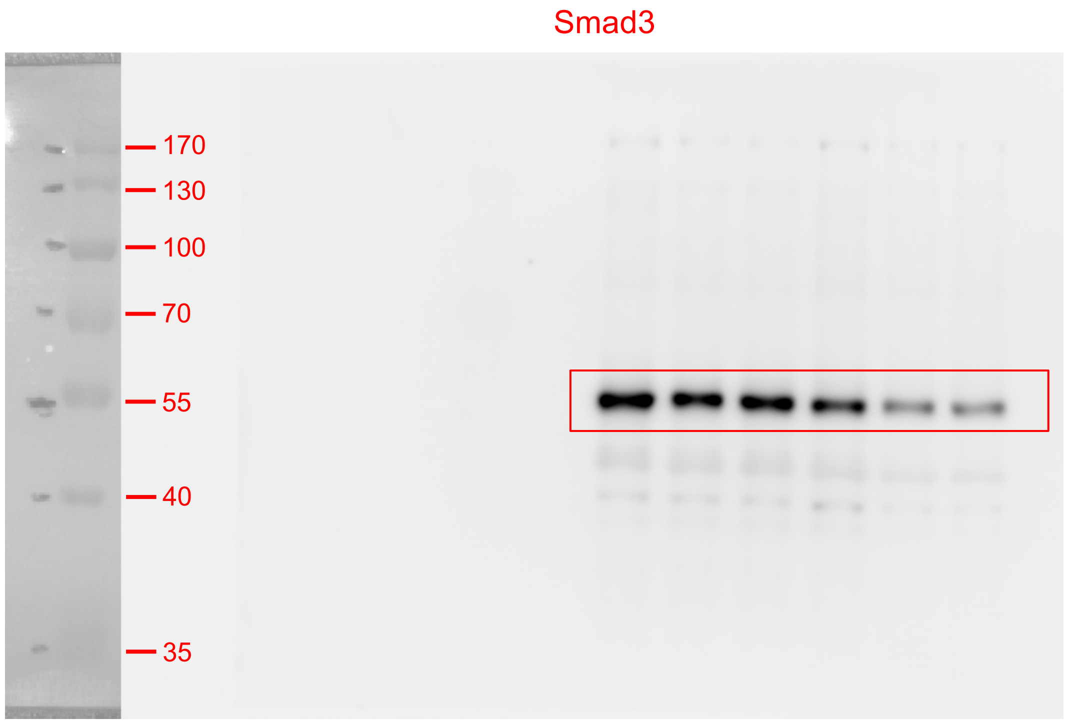

Figure 6G
Boxes highlight lanes used in the figure

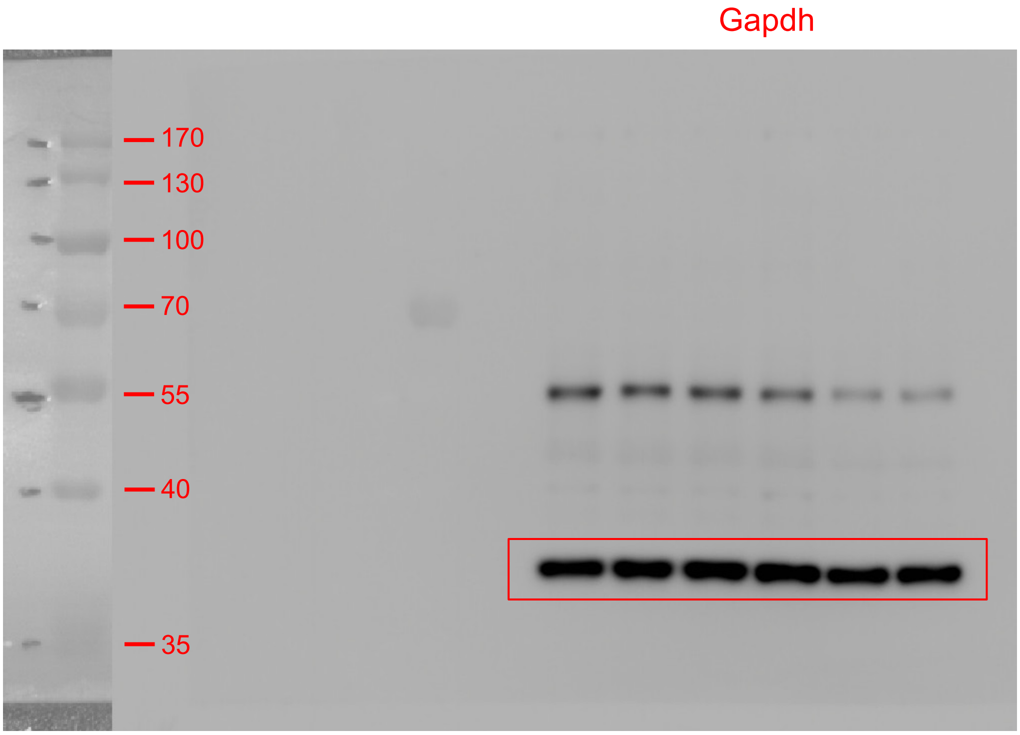

Figure 6H
Boxes highlight lanes used in the figure

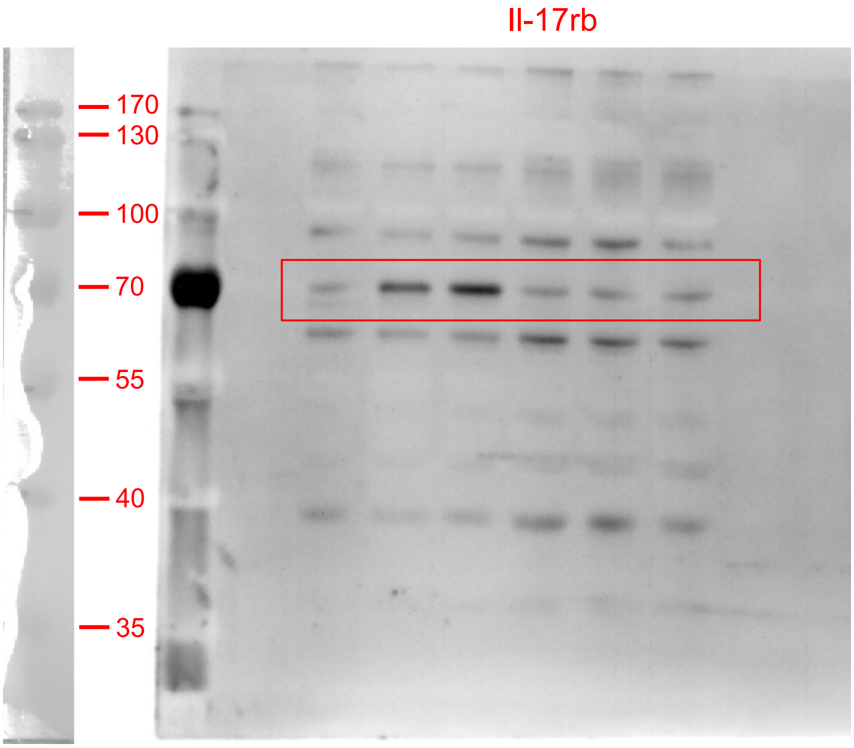

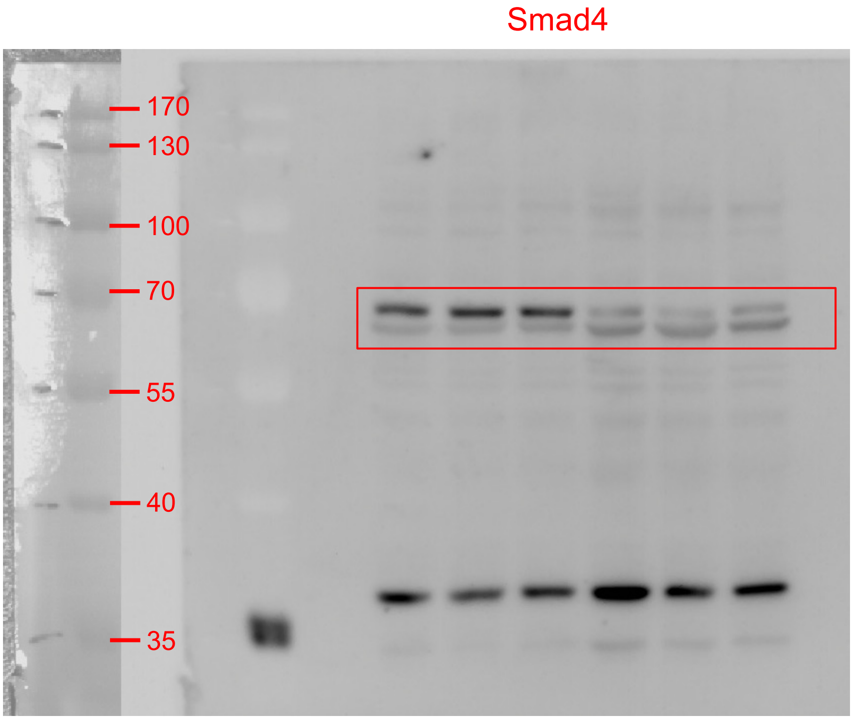

Figure 6H
Boxes highlight lanes used in the figure

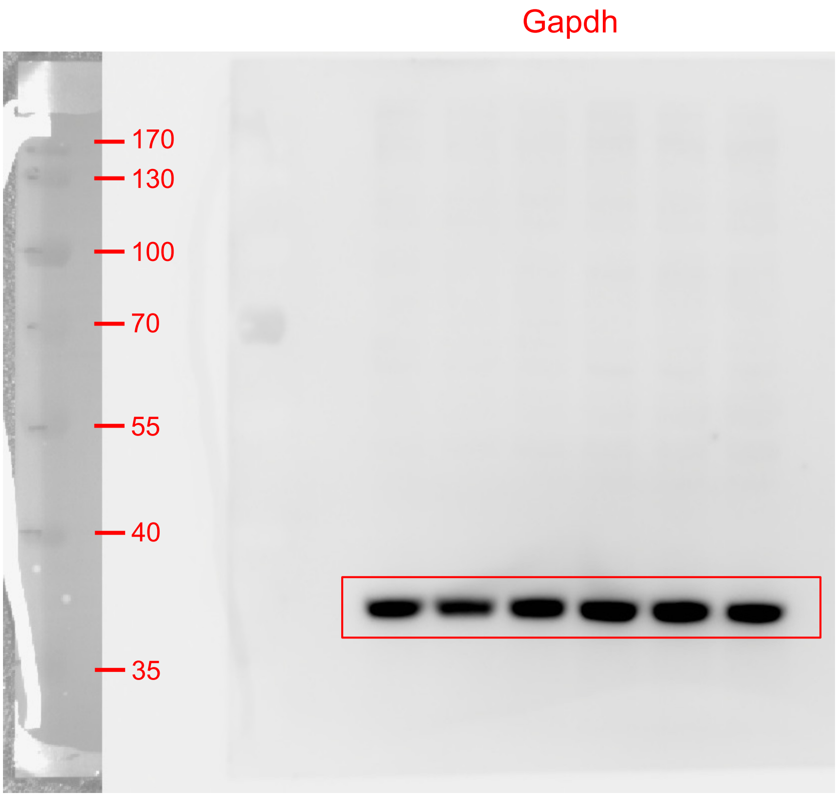

Figure 6I
Boxes highlight lanes used in the figure

NF-κB p65

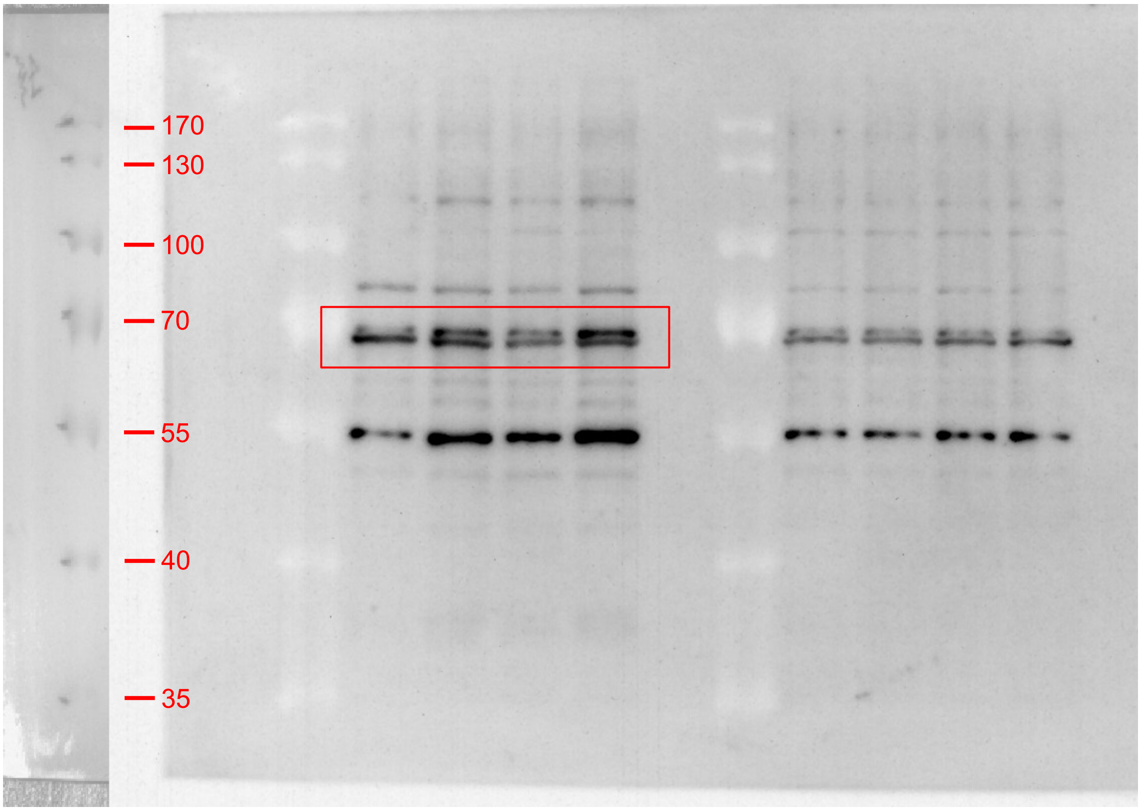

p84

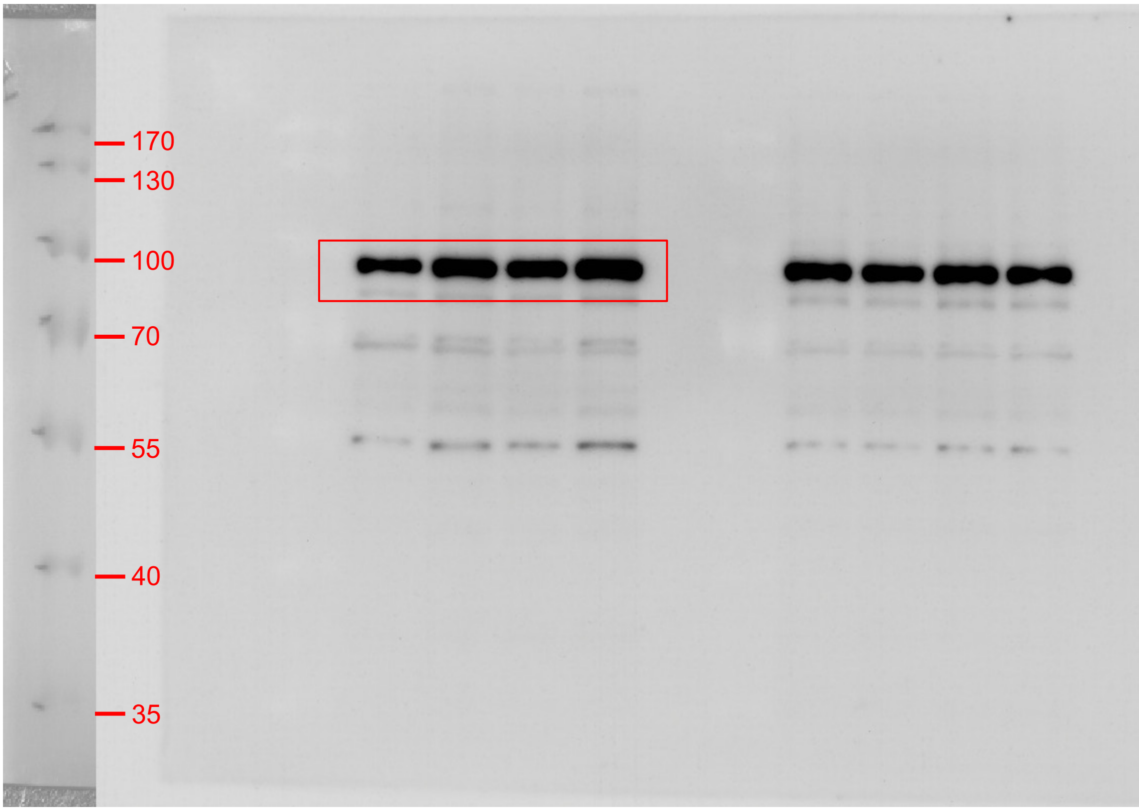

Figure 6J
Boxes highlight lanes used in the figure

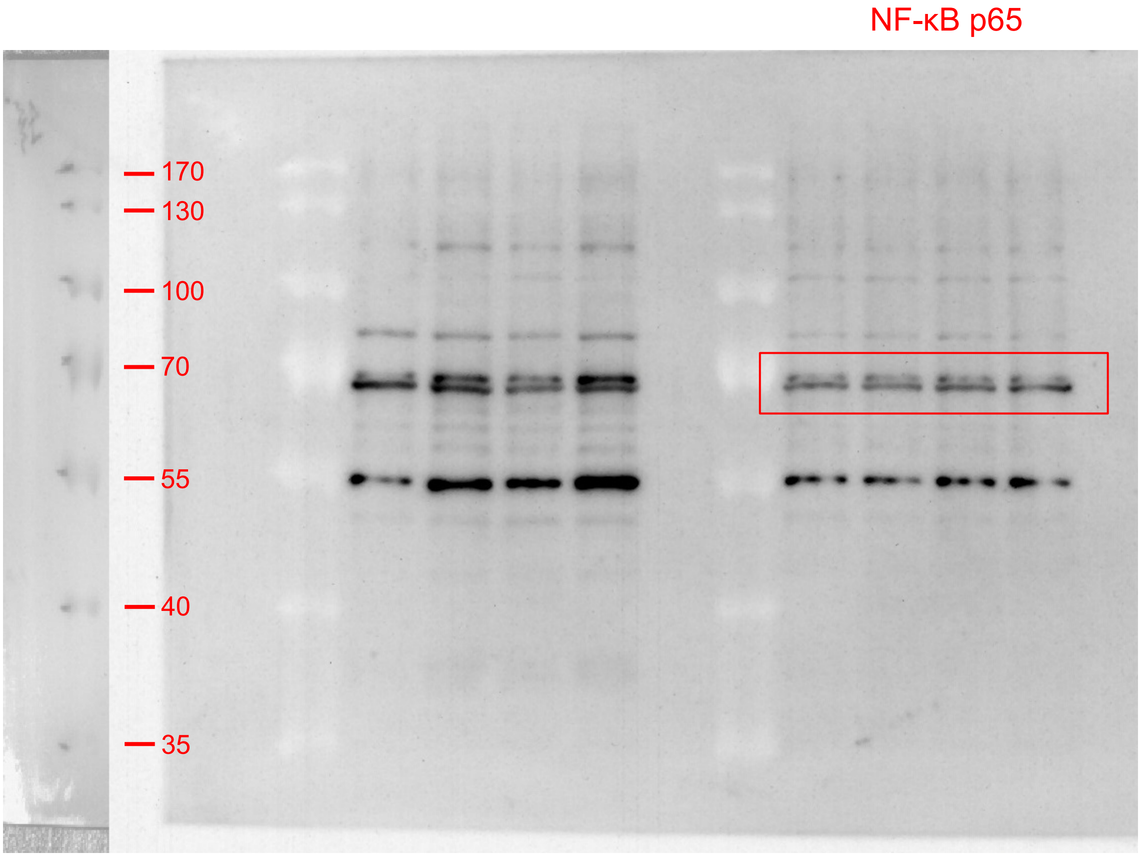

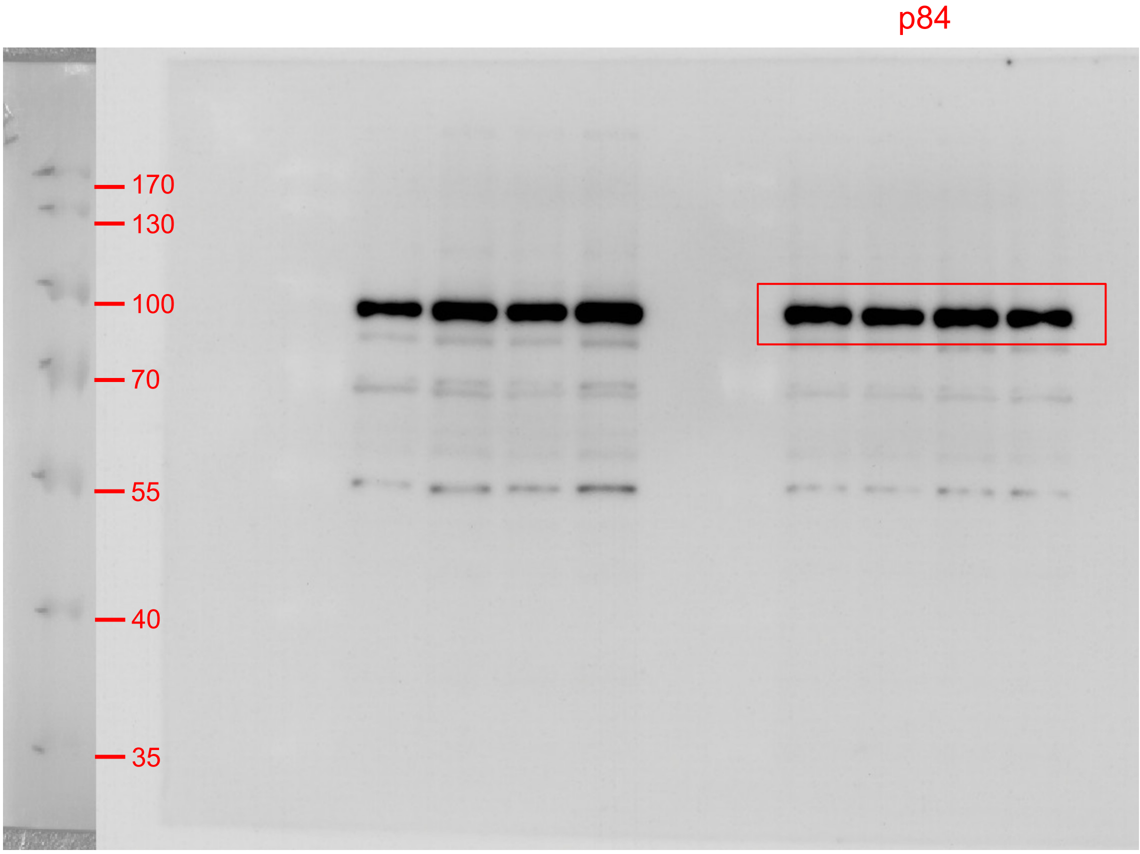

Supplement: Supplementary file 9 — Source Data for Figure 6 [file EMMM-9-1660-s008.pdf]
