# Supplementary material for: TGF‐β1 secreted by Tregs in lymph nodes promotes breast cancer malignancy via up‐regulation of IL‐17RB
Source: EMBO Mol Med. 2017 Oct 9;9(12):1660–80. doi: 10.15252/emmm.201606914 (PMC5709760; doi:10.15252/emmm.201606914)

Figure EV5A
Boxes highlight lanes used in the figure

Smad2/3
(short expo)

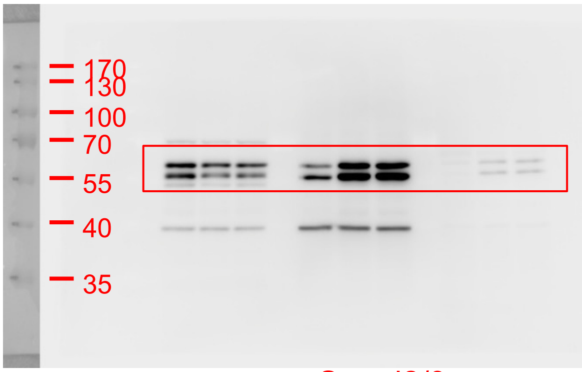

p84

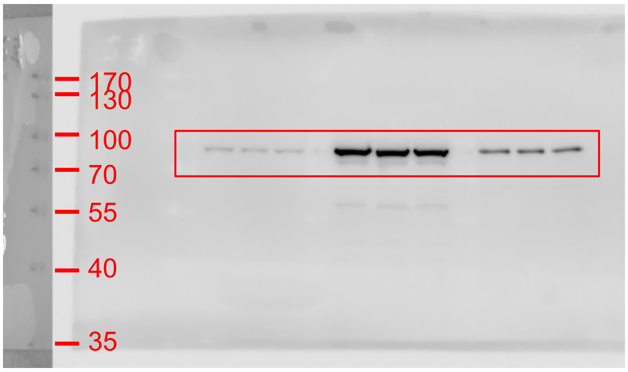

Smad2/3
(long expo)

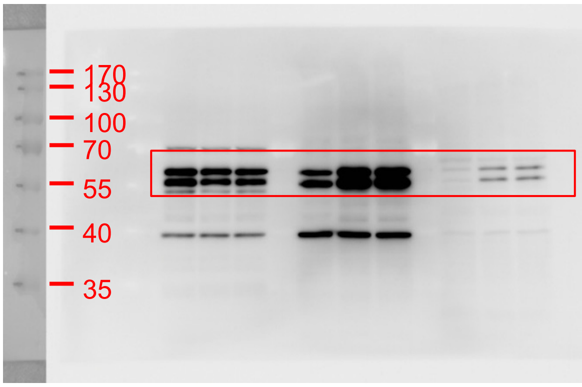

Gapdh

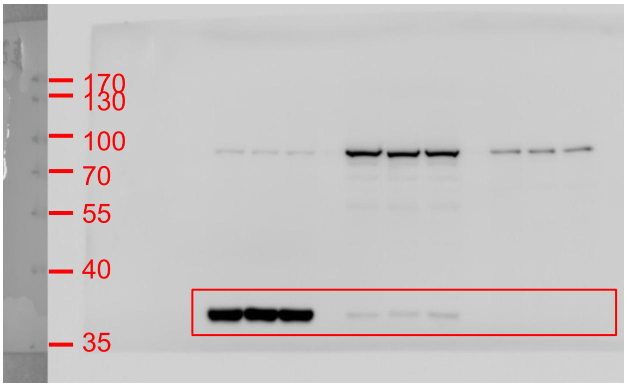

Smad6

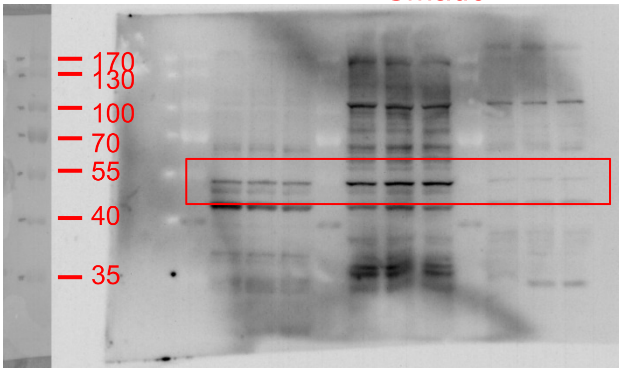

Figure EV5B
Boxes highlight lanes used in the figure

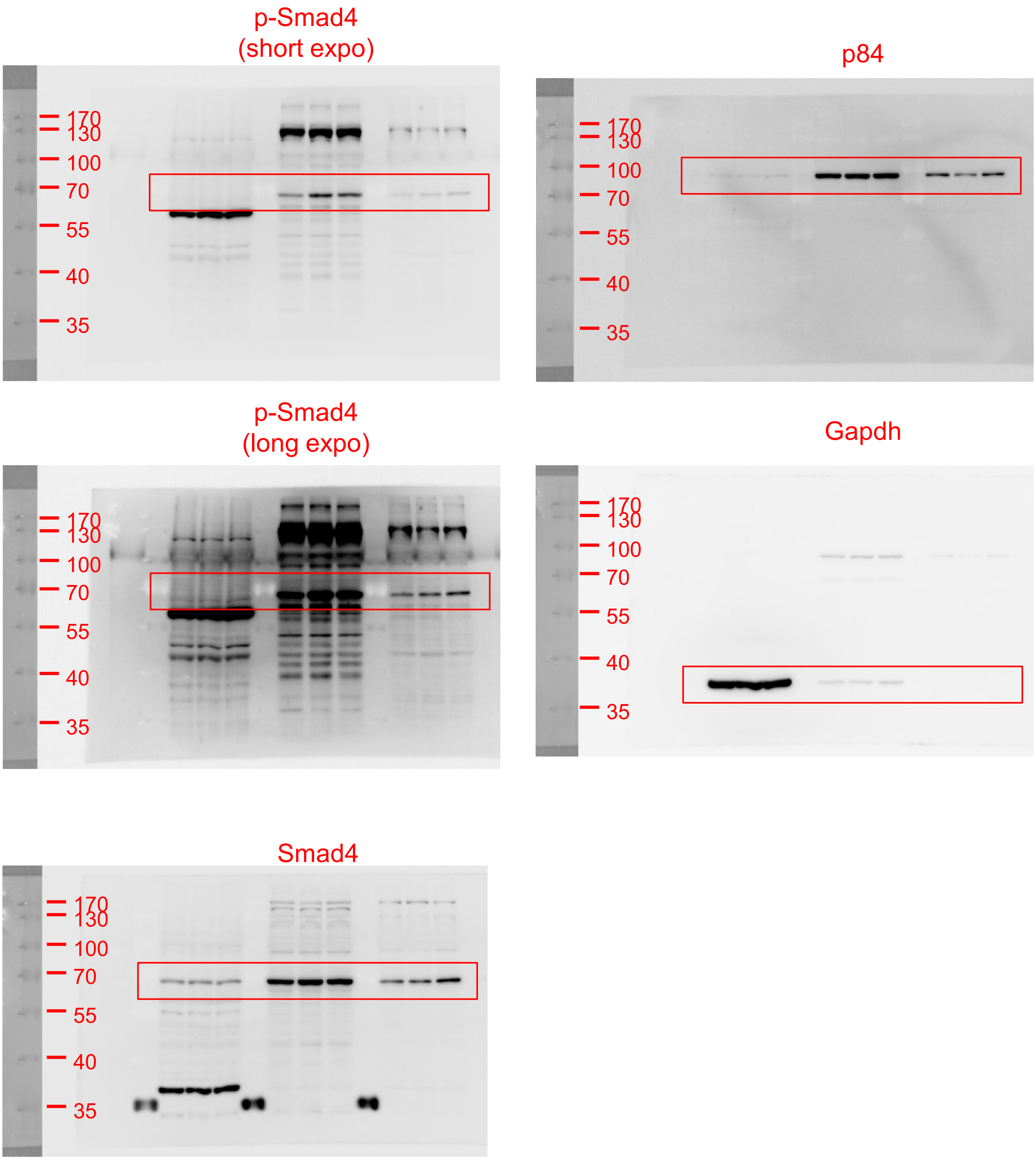

Supplement: Supplementary file 10 — Source Data for Expanded View and Appendix [file EMMM-9-1660-s009.zip › EMM06914source_dataEVandAppendix/EMM-06914-source_data/Fig_EV5_source_data.pdf]
